# Finemap-MiXeR: A variational Bayesian approach for genetic finemapping

**DOI:** 10.1371/journal.pgen.1011372

**Published:** 2024-08-15

**Authors:** Bayram Cevdet Akdeniz, Oleksandr Frei, Alexey Shadrin, Dmitry Vetrov, Dmitry Kropotov, Eivind Hovig, Ole A. Andreassen, Anders M. Dale

**Affiliations:** 1 Centre for Precision Psychiatry, Institute of Clinical Medicine, University of Oslo, Oslo, Norway; 2 Centre for Bioinformatics, Department of Informatics, University of Oslo, Oslo, Norway; 3 Constructor University Bremen, Bremen, Germany; 4 Department of Tumor Biology, Institute for Cancer Research, Oslo University Hospital, Oslo, Norway; 5 Center for Multimodal Imaging and Genetics, University of California San Diego, California, United States of America; Newcastle University, UNITED KINGDOM OF GREAT BRITAIN AND NORTHERN IRELAND

## Abstract

Genome-wide association studies (GWAS) implicate broad genomic loci containing clusters of highly correlated genetic variants. Finemapping techniques can select and prioritize variants within each GWAS locus which are more likely to have a functional influence on the trait. Here, we present a novel method, Finemap-MiXeR, for finemapping causal variants from GWAS summary statistics, controlling for correlation among variants due to linkage disequilibrium. Our method is based on a variational Bayesian approach and direct optimization of the Evidence Lower Bound (ELBO) of the likelihood function derived from the MiXeR model. After obtaining the analytical expression for ELBO’s gradient, we apply Adaptive Moment Estimation (ADAM) algorithm for optimization, allowing us to obtain the posterior causal probability of each variant. Using these posterior causal probabilities, we validated Finemap-MiXeR across a wide range of scenarios using both synthetic data, and real data on height from the UK Biobank. Comparison of Finemap-MiXeR with two existing methods, FINEMAP and SuSiE RSS, demonstrated similar or improved accuracy. Furthermore, our method is computationally efficient in several aspects. For example, unlike many other methods in the literature, its computational complexity does not increase with the number of true causal variants in a locus and it does not require any matrix inversion operation. The mathematical framework of Finemap-MiXeR is flexible and may also be applied to other problems including cross-trait and cross-ancestry finemapping.

## Author summary

Genome-Wide Association Studies report the effect size of each genomic variant as summary statistics. Due to the correlated structure of the genomic variants, it may not be straightforward to determine the actual causal genomic variants from these summary statistics. Finemapping studies aim to identify these causal SNPs using different approaches. Here, we presented a novel finemapping method, called Finemap-MiXeR, to determine the actual causal variants using summary statistics data and weighted linkage disequilibrium matrix as input. Our method is based on Variational Bayesian inference on MiXeR model and Evidence Lower Bound of the model is determined to obtain a tractable optimization function. Afterwards, we determined the first derivatives of this Evidence Lower Bound, and finally, Adaptive Moment Estimation is applied to perform optimization. Our method has been validated on synthetic and real data, and similar or better performance than the existing finemapping tools has been observed.

## Introduction

Genome-wide association studies (GWAS) have discovered hundreds of genomic loci associated with complex human traits and disorders [1]. GWAS test for association between genomic variants called single nucleotide polymorphisms (SNPs) and the corresponding traits of interest. The results of a GWAS are available as summary statistics including association effect size, standard error, and statistical significance (z-scores or p-values) for each SNP. While many SNPs may show a significant association, most of them are likely to be driven by linkage disequilibrium (LD), i.e. through correlation with a neighboring causal variant rather than through having a direct functional influence on the trait [2]. Causal SNPs may also be missed in GWAS due to insufficient statistical power, unmeasured or unimputed SNPs [3]. Statistical finemapping methods aim to identify causal SNPs within a given locus after controlling for LD.

There are some existing finemapping methods in the literature. Bayesian methods offer important advantages in finemapping causal SNPs compared to other heuristic and penalized regression methods, especially in situations where the true number of causal SNPs per locus is high [4]. Having multiple causal SNPs in a locus is a plausible situation that is often observed in complex human traits. For instance, it is shown that number of causal SNPs for prostate cancer ranges from 1 to 5 in different loci [5]. Similarly, for Alzheimer’s disease (ALZ), recent studies provide evidence that a large number of variants on chromosome 19 around the APOE region modify ALZ risk [6]. Most of the early finemapping methods such as BIMBAM [7], CAVIAR [8], CAVIARBF [9], and PAINTOR [10] rely on exhaustive searches of the possible causal configurations for a given locus and calculating corresponding posterior causal probabilities of each SNP. Despite the accuracy of these methods, they are computationally inefficient, especially if the number of causal variants (k) or the total number of SNPs per locus (M) is high, precluding exhaustive search across all (Mk) causal configurations.

Benner *et*. *al*. developed a computationally efficient method called FINEMAP [11] that calculates the likelihood function using Cholesky Decomposition, and then searches possible causal configurations via the Shotgun Stochastic Search [12]. Thanks to these improvements, the computational complexity has been reduced, while preserving the same accuracy as the previous Bayesian exhaustive search methods like CAVIARBF. An extension of the FINEMAP method [13] can also estimate the effect sizes of causal variants, and heritability attributed to the locus being analysed.

Another recent approach to finemapping is based on applying a modified version of Single Effect Regression model [7], called the Sum of Single effects (SuSiE) [14]. The main idea behind this method is optimizing the proposed model by eliminating the effect of each causal SNP using Iterative Bayesian stepwise selection (IBSS). Compared to the other Bayesian variable selection methods [15,16], SuSiE has lower computational complexity, and is more suitable for inference on highly correlated variables. It was demonstrated that SuSiE had better accuracy than the previously published finemapping methods [14]. While the original SuSiE algorithm requires individual-level genotype and phenotype data as input, it has been expanded to SuSiE Regression Summary Statistics (RSS) method which only requires summary statistics-level data [17]. SuSiE-RSS yields a similar accuracy as the original SuSiE algorithm, and at the same time reduces the computational complexity.

Despite the effectiveness of currently available finemapping methods, they can still be improved in terms of both accuracy and computational aspects. Here, we present a novel Finemap-MiXeR method, based on variational Bayesian approach leveraging the MiXeR model [2]. The MiXeR model assumes a biologically plausible prior distribution of SNPs and can estimate the heritability, polygenicity and discoverability of a given trait, and the polygenic overlap between two traits [18]. In Finemap-MiXeR, following variational Bayesian approach, the likelihood function of observing GWAS z-scores is replaced with its Evidence Lower Bound (ELBO). We analytically determined the derivatives of the ELBO function and optimized it with the Adaptive Moment Estimation (ADAM) algorithm [19]. This method requires summary statistics and scaled LD-matrix, and outputs the posteriors of SNPs being causal namely *posterior causal probabilities*.

Our proposed finemapping method has several advantages over existing tools. First, we show increased accuracy of Finemap-MiXeR over FINEMAP and SuSiE in detecting causal SNPs in simulation, across a broad range of scenarios. Furthermore, despite the increase in performance is limited compared to other methods, our method can also detect different causal variants that other methods did not identify in some scenarios. We also validated our method in height, Alzheimer’s disease (ALZ) and Parkinson’s disease (PD) applications. The computational complexity of Finemap-MiXeR is only increasing with respect to the number of SNPs per locus (M) and unlike other methods it is not increasing as the number of causals (k) or locus’s heritability (*h*^2^) increases. Furthermore, unlike many existing finemapping methods, Finemap-MiXeR does not require to compute the inverse of the LD matrix, which is an important aspect and is broadly considered in various studies, such as [20,21]. Finally, the flexibility of our mathematical framework provides possibilities to extend the current approach in various directions, such as finemapping in multiple traits or across ancestries (For details see Discussion section). Taken together these advantages of Finemap-MiXeR represent an important step forward in our ability to disentangle biological insights from the associations observed in GWAS.

## Description of the method

### Ethics statement

The UK Biobank was granted ethical approval by the North West Multi-centre Research Ethics Committee (MREC) to collect and distribute data and samples from the participants (http://www.ukbiobank.ac.uk/ethics/) and covers the work in this study, which was performed under UK Biobank application numbers 27412. All participants included in these analyses gave written consent to participate.

### Variational Bayesian inference on the MiXeR model

The Finemap-MiXeR method takes summary statistics and a scaled LD matrix (A) as input and, using the MiXeR model, it outputs the posterior causal probability of each SNP (*q*_*i*_), alongside with the expectancy of effect size of the SNP (*μ*_*i*_) and uncertainty ($\sigma_{i}^{2}$) of the effect size estimate. This is achieved by applying variational Bayesian inference on the MiXeR model. For more details of mathematical aspects, see S1 Notes.

In the MiXeR model [2], we can write the z-score of j-th SNP (*z*_*j*_) as a linear combination of the ground-truth effect sizes of all SNPs, and the coefficients comes from the scaled version of the LD matrix, namely A. Given *a*_*ij*_ as the element of matrix A for i-th row and j-th column, it can be written as derived in [22]:

$$a_{ij}=\sum_{i=1}^{M}\sqrt{{N\hat{H}}_{i}}{\hat{r}}_{ji}$$

where N is sample size, ${\hat{H}}_{i}$ is estimated SNP’s heterozygosity (${\hat{H}}_{i}=2f_{i}(1-f_{i})$ where *f*_*i*_ is minor allele frequency of i-th SNP) and ${\hat{r}}_{ji}$ is estimated correlation coefficient between SNPs i and j. Having obtained A matrix, we can write *z*_*j*_ as:

$$z_{j}=\sum_{i=1}^{M}a_{ij}\beta_{i}+e_{j},$$

where *e*_*j*_ is error term and typically modelled as a Gaussian distribution as $N(e_{j}|0,\sigma_{0}^{2})$.

The MiXeR model assumes only a fraction of all SNPs (*π*_1_) in a locus are causal (i.e., have a non-zero ground-truth effect size *β*_*i*_) for a given phenotype, and can be postulated using a spike and slab prior as:

$$p(\beta_{i})=(1-\pi_{1})N(\beta_{i}|0,\delta^{2})+\pi_{1}N(\beta_{i}|0,\sigma_{\beta }^{2}),$$

where *π*_1_∈[0,1] indicates the weight in the Gaussian mixture, *N*(.|,) denotes a normal distribution (*N*(.|0,*δ*^2^) is a normal distribution with sufficiently small variance *δ*^2^ to represent spike distribution), and $\sigma_{\beta }^{2}$ is a parameter of MiXeR model to represent the variance of non-zero effects and these parameters can be written as

$$h^{2}=\sigma_{\beta }^{2}\pi_{1}\sum_{i=1}^{M}{\hat{H}}_{i}.$$

In this work, we assume that parameters $\theta =(\pi_{1},\sigma_{\beta }^{2},\sigma_{0}^{2})$ are the same across all SNPs, i.e., do not depend on *i*. It is also possible to expand our proposed approach for SNPs with individual priors in future work on the model.

To determine the likelihood of the MiXeR model, we introduce the latent variables *u*_*i*_∈{0,1} following Bernoulli distribution, *p*(*u*_*i*_) = *Bern*(*u*_*i*_|*π*_1_). Then the full probabilistic model is p(z,β,u|θ)=p(z|β,θ)·p(β|u,θ)·p(u|θ)), explicitly written as

$$\begin{array}{ll} & p(z_{j}|\beta_{1},\ldots ,\beta_{M},\theta )=N(z_{j}|\sum_{i=1}^{M}a_{ij}\;\beta_{i},\sigma_{0}^{2}),\\ & p(\beta_{i}|u_{i}=0,\theta )=N(\beta_{i}|0,\delta^{2}),\;p(\beta_{i}|u_{i}=1,\theta )=N(\beta_{i}|0,\sigma_{\beta }^{2}),\\ & p(u_{i}|\theta )=Bern(u_{i}|\pi_{1}). \end{array}$$

After observing z = (*z*_1_,…,*z*_*M*_)^*T*^, we can do inference on *θ* by maximum likelihood as

$$p(z|\theta )=\int_{u}\int_{\beta }p(z,\beta ,u|\theta )dud\beta .$$

Note that numeric optimization of the above *p*(*z*|*θ*) expression is not tractable, however it can be replaced with its Variational Lower Bound:

$$\begin{array}{l} \log\;p(z|\theta )=E_{q(\beta ,u)}[\log\;p(z,\beta ,u|\theta )-\log\;q(\beta ,u)]+KL(q(\beta ,u)||p(\beta ,u|z,\theta ))\geq \\ \;\geq E_{q(\beta ,u)}[\log\;p(z,\beta ,u\theta )-\log\;q(\beta ,u)]=L(q,\theta ), \end{array}$$

where ℒ(*q*,*θ*) is the variational lower bound of log *p*(*z*|*θ*), *q*(*β*,*u*) is a distribution function from any parametric family and KL(.||.) is Kullback-Leibler divergence operator. Choosing *q*(*β*,*u*) to be close to the *p*(*β*,*u*|*z*,*θ*) distribution leads to low values of the KL(q(β,u)||p(β,u|z,θ) term, thus making ℒ(*q*,*θ*) a tight lower bound. In this situation the optimization of *p*(*z*|*θ*) will be almost equivalent to the optimization of ℒ(*q*,*θ*) (in a sense that any local maximum of the second problem will also yield local maximum of the original optimization problem).

We will search *q*(*β*,*u*) from the following parametric family:

$$q(\beta ,u)=\prod_{i=1}^{M}Bern(u_{i}|q_{i})N(\beta_{i}|\mu_{i},\sigma_{i}^{2}).$$

Using this model and parametric family, we can optimize ℒ(*q*,*θ*) and obtain the parameters of the *q*(*β*,*u*) which corresponds to the posterior causal probability of each SNP (q_i_), and parameter (*μ*_*i*_) indicating corresponding effect size. Note that we use the same parametric family *q*(*β*,*u*) as proposed in [23], that applied variational Bayesian approach for Polygenic Risk Score (PRS) analysis. Our method is different in that we proceed with derive derivatives of the ELBO function using its derivatives, as an alternative to variational EM algorithm used in [23]. Also, our application specifically focused on accuracy of finemapping causal variants and developed accordingly, rather than genome-wide polygenic risk prediction.

### Derivation of derivatives of ELBO function

In order to perform the optimization of ℒ(*q*,*θ*), we will be using the Adaptive moment estimation (ADAM) algorithm, which computes the adaptive learning rate for each parameter using the first derivatives of ℒ(*q*,*θ*). Therefore, we need to calculate the corresponding derivatives with respect to *μ*_*i*_, *σ*_*i*_ and *q*_*i*_ analytically.

For this aim, we expanded ℒ(*q*,*θ*) and then perform various mathematical treatments including the Reparameterization trick [24]. Firstly, we may expand ELBO function, ℒ(*q*,*θ*), as

$$\begin{array}{l} L(q,\theta )=E_{q(\beta ,u)}\log p(z|\beta ,\theta )+E_{q(\beta ,u)}\log\frac{p(\beta |u,\theta )p(u|\theta )}{q(\beta ,u)}=\\ =E_{q(\beta )}\log p(z|\beta ,\theta )-E_{q(u)}\sum_{i=1}^{M}KL(q(\beta_{i})||p(\beta_{i}|u_{i},\theta ))-\sum_{i=1}^{M}KL(q(u_{i})||p(u_{i}|\theta )). \end{array}$$

We, then define these three terms of ℒ(*q*,*θ*) as ℒ(*q*,*θ*) = *T*_1_−*T*_2_−*T*_3_, where *T*_1_, *T*_2_ and *T*_3_ are defined as follows:

$$T_{1}=E_{q(\beta )}\sum_{j=1}^{M}\log p(z_{j}|\beta ,\theta ),$$

$$T_{2}=E_{q(u)}\sum_{i=1}^{M}KL(q(\beta_{i})||p(\beta_{i}|u_{i},\theta )),$$

$$T_{3}=\sum_{i=1}^{M}KL(q(u_{i})||p(u_{i}|\theta )).$$

Then we applied various mathematical techniques to calculate the derivatives of *T*_1_, *T*_2_ and *T*_3_. These approaches are presented in S1 Notes. In the end we obtained the derivatives of the ℒ(*q*,*θ*), $\frac{\partial L_{q,\theta }}{\partial \mu_{i}},\frac{\partial L_{q,\theta }}{\partial \sigma_{i}^{2}},\frac{\partial L_{q,\theta }}{\partial q_{i}}$ and also derivative of hyperparameters, $\theta =(\pi_{1},\sigma_{\beta }^{2},\sigma_{0}^{2})$ as given in Table 1. We will use these derivatives with ADAM algorithm to optimize ELBO. (For the details of the implementation of ADAM algorithm see S1 Notes.)

**Table 1:** All partial derivatives of ℒ_*q*,*θ*_. *T*_*A*_ is a function of *a*_*ij*_ and *z*_*j*_. For details see S1 Notes.

$\frac{\partial L_{q,\theta }}{\partial \mu_{i}}$	$\frac{1}{\sigma_{0}^{2}}\sum_{j=1}^{M}a_{ij}(z_{j}-\sum_{k=1}^{M}a_{kj}\mu_{k})-\frac{(1-q_{i})\mu_{i}}{\delta^{2}}-\frac{(q_{i})\mu_{i}}{\sigma_{\beta }^{2}}$
$\frac{\partial L_{q,\theta }}{\partial \sigma_{i}^{2}}$	$\frac{-1}{4\sigma_{0}^{2}}\sum_{j=1}^{M}2a_{ij}^{2}-\frac{1}{2}(\frac{\left(1-q_{i}\right)}{\delta^{2}}+\frac{\left(q_{i}\right)}{\sigma_{\beta }^{2}}-\frac{1}{\sigma_{i}^{2}})$
$\frac{\partial L_{q,\theta }}{\partial q_{i}}$	$‐\left(\log(\frac{\sigma_{\beta }}{\delta })-\frac{\sigma_{i}^{2}+\mu_{i}^{2}}{2\delta^{2}}+\frac{\sigma_{i}^{2}+\mu_{i}^{2}}{2\sigma_{\beta }^{2}}+\log\frac{q_{i}}{\pi_{1}}-\log\frac{1-q_{i}}{1-\pi_{1}}\right)$
$\frac{\partial L_{q,\theta }}{\partial \pi_{1}}$	$\sum_{i=1}^{M}\frac{\pi_{1}-q_{i}}{\pi_{1}-\pi_{1}^{2}}$
$\frac{\partial L_{q,\theta }}{\partial \sigma_{\beta }^{2}}$	$\sum_{i=1}^{M}\frac{q_{i}}{2\sigma_{\beta }^{4}}(\sigma_{i}^{2}+\mu_{i}^{2}-\sigma_{\beta }^{2})$
$\frac{\partial L_{q,\theta }}{\partial \sigma_{0}^{2}}$	$\frac{T_{A}-M\sigma_{0}^{2}}{2\sigma_{0}^{4}}$

### Hyperparameters

As stated before, we assumed that all hyperparameters of the MiXeR model $\theta =(\pi_{1},\sigma_{\beta }^{2},\sigma_{0}^{2})$ are the same across all SNPs. Those parameters can either be selected by user if the ground truth values of them are known ($\sigma_{0}^{2}$ can be obtained from (2), *π*_1_ is defined by user and $\sigma_{\beta }^{2}$ is estimated via using $h^{2}=\sigma_{\beta }^{2}\pi_{1}\sum_{i=1}^{M}{\hat{H}}_{i}$.) or can also be optimized during the ADAM algorithm using the derivatives presented in Table 1. In Fig A in S1 Text, we refer to the latter option as “Finemap-MiXeR with optimizing hyperparameters”, and to the former option as Finemap-MiXeR. As shown in Fig A in S1 Text, both methods give almost identical results. In the following experiments and simulations, we used Finemap-MiXeR with optimizing hyperparameters but recall it as Finemap-MiXeR for the sake of simplicity.

### Credible sets

Credible sets are frequently used in finemapping literature to define a set of SNPs that includes a causal SNP with a given probability [25]. Since many loci have complex LD structure, it is also important to report such credible sets in order not to miss possible causal SNPs. For instance, a finemap method may report two SNPs as causal with non-negligible posterior causal probabilities and if the correlation among these SNPs is high, it is beneficial to report both of these SNPs in a credible set. Our method is also capable of reporting such credible sets. A credible set namely CS_U_(Q_u_,q_thr,_ *η*), can be constructed using the SNPs listed by *U* and satisfies the following two constraints:

$${i)\;P}_{k}=\sum_{i\epsilon U}\;q_{i}>q_{thr}$$

$$ii)\;{min}_{i,j\epsilon U}\;r_{i,j}>\eta$$

where Q_U_ = {q_i,_ such that *i* ϵ *U*} and *η* is the purity threshold, i.e. the smallest allowed absolute correlation threshold among variants within a credible set. For loci with multiple possible causal SNPs, it is expected to report multiple credible sets where each set includes one causal SNP as suggested in SuSiE. In our method, these credible sets can be constructed considering corresponding correlations among SNPs. In other words, SNPs with highest posterior causal probabilities construct a credible set based on desired smallest allowed absolute correlation threshold (purity). For a given purity threshold within a credible set, *η*, L candidate credible sets can be constructed using posterior causal probabilities as:

Step 1. Sort obtained posterior causal probabilities (*q*_*i*_) in descending order. Let Q be the list of these sorted variants.

Step 2. Assign L candidate credible sets by choosing L variants that have highest *q*_*i*_ and their pairwise absolute correlation is lower than *η*

Step 3. Add more variants to these sets from the list of Q whose pairwise absolute correlation is higher than *η*. Remove these added variant from the list of Q.

Step 4. Repeat Step 3 for each set until it satisfies *P*_*k*_ > q_thr_

Step 5. Discard sets who do not satisfy *P*_*k*_ > q_thr,_ and report the resulting L* credible sets where L*≤*L*.

Following this procedure, we can report multiple credible sets that include variants whose absolute correlation is greater than *η* and satisfy *P*_*k*_ > q_thr_. The choice of the initial number of credible sets (L) is not required to be determined by the user. Since hyperparameters can be optimized during the finemapping procedure, we can obtain an optimized π_1_, which implies that the number of causal variants would be M π_1_. Therefore, having a higher number of L than this number (in our simulations, we chose L = ⌈ M π_1_⌉, where ⌈.⌉ is the ceiling operator) will be sufficient to be able to capture all possible credible sets. Furthermore, L could also be chosen as any number bigger than M π_1_, and it is observed that the results are not sensitive to the choice of L for the 0.95 credible set threshold, given that it is bigger than the number of causals (See Fig E in S1 Text for details). This is expected, since having a bigger L may initially construct a larger number of credible sets. However, eventually the redundant credible sets would be eliminated at step 5. Having a lower number of L than M π_1_ may however lead to missing some possible credible sets.

The choice of q_thr_ and *η* also affect the number of possible credible sets. Therefore, if q_thr_ is chosen as lower than the conventional threshold (0.95), we may expect a higher number of credible sets (in such cases, L can be internally and automatically adjusted to a higher number (higher than *L =* ⌈ *M* π_1_⌉), depending on the chosen q_thr_). Similarly, if *η* is chosen too low, it may lead to encompassing two true causal variants in the same credible set if their absolute correlation is greater than *η*. This may result in having a credible set with two (or more) true causals, thus reducing the number of credible sets.

### Computational complexity of Finemap-MiXeR

In Finemap-MiXeR, ADAM Algorithm is used to optimize ELBO. As mentioned before, in ADAM algorithm, it is required to calculate the first derivatives of the parameters of interest for each iteration. Therefore, computational complexity of Finemap-MiXeR depends on the computational cost of the calculation of these derivatives per iteration. To calculate this, we will examine the derivatives one by one, starting with $\frac{\partial L_{q,\theta }}{\partial \mu_{i}}$:

$$\frac{\partial L_{q,\theta }}{\partial \mu_{i}}=\left(\frac{1}{\sigma_{0}^{2}}\sum_{j=1}^{M}a_{ij}(z_{j}-\sum_{k=1}^{M}a_{kj}\mu_{k})\right)-\frac{(1-q_{i})\mu_{i}}{\delta^{2}}-\frac{(q_{i})\mu_{i}}{\sigma_{\beta }^{2}}.$$

Note that $\frac{\partial L_{q,\theta }}{\partial \mu_{i}}$ can be written in more compact from as

$$\frac{\partial L_{q,\theta }}{\partial \mu }=\frac{1}{\sigma_{0}^{2}}(A_{1}+A_{2}\mu {)}^{T}-\frac{(1-q)⊙\mu }{\delta^{2}}-\frac{q⊙\mu }{\sigma_{\beta }^{2}},$$

where *A*_1_ = *A***z** and *A*_2_ = −*AA*^*T*^, ⊙ is Hadamard product (element-wise multiplication), and **q** and **μ** are the vectors that have all *q*_*i*_ and *μ*_*i*_ elements, respectively. Since *A*_1_ and *A*_2_ can be pre-computed, the required computation per iteration is *O*(*M*^2^) which comes from *A*_2_**μ** term (the Hadamard product operations require *O*(M) hence can be neglected). From Table 1, one can also observe that the computational complexity of calculating $\frac{\partial L_{q,\theta }}{\partial \sigma_{i}^{2}}$ and $\frac{\partial L_{q,\theta }}{\partial q_{i}}$ are both *O*(*M*). Therefore total computational complexity of the algorithm per iteration is *O*(*M*^2^).

### Reducing computational complexity with Finemap-MiXeR-PCA

As discussed above, the computational complexities of calculating $\frac{\partial L_{q,\theta }}{\partial \mu_{i}},\frac{\partial L_{q,\theta }}{\partial \sigma_{i}^{2}},\frac{\partial L_{q,\theta }}{\partial q_{i}}$ derivatives are *O*(*M*^2^), *O*(*M*) and *O*(*M*) respectively. Hence, if we can somehow reduce the computational complexity of $\frac{\partial L_{q,\theta }}{\partial \mu_{i}}$, we will also reduce the computational complexity of the whole algorithm. We present a Principal Component Analysis (PCA) based approach, namely Finemap-MiXeR PCA, to reduce the computational complexity as presented in S1 Notes. For calculating $\frac{\partial L_{q,\theta }}{\partial \mu_{i}},$ there is only one term that requires *O*(*M*^2^) and it is *A*_2_**μ** which is a MxM matrix and Mx1 vector product where *A*_2_ = −*AA*^*T*^. Using PCA, it is possible to calculate this term with O(*p*_*c*_ M) where *p*_*c*_ is mostly *p*_*c*_<<*M*, the required operations to compute gradients can be reduced importantly by preserving accuracy. Although PCA itself requires some computations to be done before implementing ADAM algorithm, this is calculated one time and there is not any need to recalculate it during the optimization. Also, it is possible to reduce PCA calculation cost with some efficient algorithms such as Lanczos algorithm [26].

### Verification and comparison

We compared Finemap-MiXeR method with FINEMAP (FINEMAP 1.4) and SuSiE RSS in terms of their accuracy and runtime performance. Using synthetic data with known location of causal variants the accuracy of the methods was measured using the area under Receiver Operating Characteristic (ROC). When comparing the methods using real data on height from UK Biobank (UKB), the true location of causal variants are unknown, and we therefore used a proxy measure of finemapping accuracy, evaluating how well we could predict the phenotype from SNPs selected as causal by each of the methods. For the runtime performance we also compared Finemap-MiXeR-PCA and SuSiE in the “Runtime Performance and Computational Complexity” section below, but omitted their performances on accuracy, since their performances in terms of accuracy were almost identical with Finemap-MiXeR and SuSiE RSS respectively (See Fig A in S1 Text). Note that for all experiments, exactly same data is used for all methods for the sake of fair comparison. We also applied our Finemap-MiXeR method to Alzheimer’s disease and Parkinson’s disease. Note that, for synthetic data, we have assigned the causal variants and simulate the phenotype accordingly, while for height, Alzheimer’s disease (ALZ) and Parkinson’s disease (PD), we have chosen loci with at least one SNP strongly associated with a trait, and then applied these loci to finemapping as defined elsewhere [4].

### Simulation with synthetic data

The first scheme is evaluation of the performances with synthetic genotype data with realistic LD structure, created using the Hapgen2 tool [27], and simulating the phenotypes by arbitrarily choosing the actual causal SNPs with pre-defined true heritability in a given locus. Given an NxM genome matrix (G) for N “subjects” and M SNPs, a phenotype vector with a desired heritability within the locus (*h*^2^) was obtained by randomly pre-assigning the causal SNPs with a vector *β* (where *β*_i_ = 1 if the SNP is causal and 0 otherwise) as

$$y=c_{1}\;G\beta +c_{2}\epsilon$$

where $c_{1}=\sqrt{\frac{h^{2}}{var(G\beta )}}$ and $c_{2}=\sqrt{\left(1-h^{2}\right)}$ and *ϵ* is a residual following standard normal distribution.

**Fig 1:**
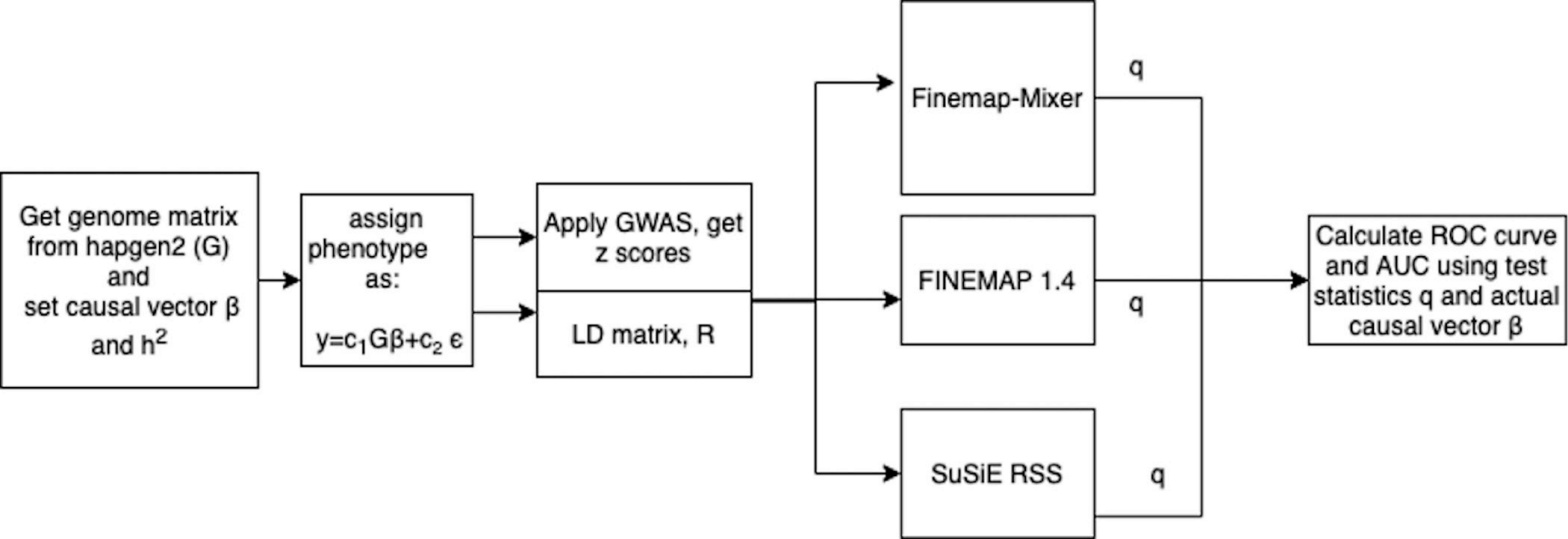
Overview of the steps used for validation of the Finemap-MiXeR method with synthetic data. Firstly, we randomly selected a locus containing pre-defined number (M) of adjacent SNPs, randomly selected “k” causal variants within the loci, and draw their effect sizes (vector *β*). Then, we used synthetic genotype data (G) with realistic LD structure, as generated by hapgen2 tool, to calculate the phenotypic values (y) for all individuals using additive genetic model (*y* = *c*_1_*Gβ*+*c*_2_*ϵ*), where scaling constants *c*_1_ and *c*_2_ were chosen to yield *Var*(*y*) = 1 and *Var*(*c*_1_*Gβ*) = *h*^2^ (pre-defined value indicating true heritability of the loci). Using G and y we calculated z-scores by applying GWAS, and then used them as inputs for the tools to obtain posterior causal probabilities of each SNP. Since we know ground truth (the location of causal variants), we then determined Receiver Operating Characteristic (ROC) curves for Finemap-MiXeR and the comparison methods (SuSiE RSS and FINEMAP 1.4) and calculated corresponding Area Under the Curve (AUC).

Using the procedure described above and sketched in Fig 1, we randomly chose a locus from this synthetic genome data and obtained the corresponding G matrix and then determined the artificial phenotype vector y for different values of M and *h*^2^ for N = 10 000. This procedure is repeated 50 times in each scenario particularly for different numbers of causals. We have also repeated same simulation procedure by randomly pre-assigning the causal SNPs with a vector *β* with normal distribution where *β*_i_ = N(0,1) if the SNP is causal and 0 otherwise.

**Fig 2:**
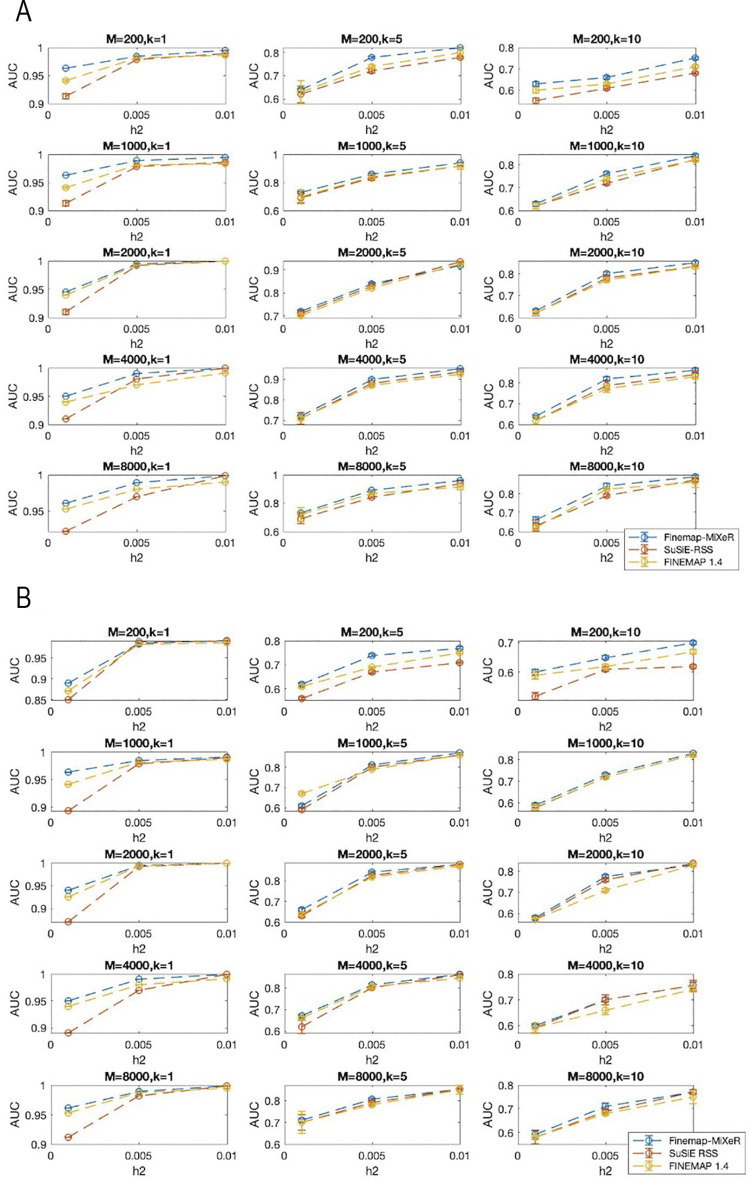
**(A)** Area Under the ROC Curve (AUC) comparison of Finamep-MiXeR with SuSiE RSS and FINEMAP across different scenarios, varying: the size of the locus being analyzed (M = 200, 1000, 2000, 4000, or 8000 SNPs per locus, shown in rows); the true number of causal variants (k = 1, 5, or 10, shown in columns), and the true heritability within the locus (h2 = 0.001, 0.005, or 0.01, shown on the horizontal axis of each panel). Effect size of causal SNPs are randomly assigned as *β*_i_ = 1 and then adjusted based on given heritability. The curves represent Receiver Operating Characteristic (ROC) curve averaged across 50 different simulations with corresponding standard error. **(B)** Area Under the ROC Curve (AUC) comparison of Finamep-MiXeR with SuSiE RSS and FINEMAP across different scenarios, varying: the size of the locus being analyzed (M = 200, 1000, 2000, 4000, or 8000 SNPs per locus, shown in rows); the true number of causal variants (k = 1, 5, or 10, shown in columns), and the true heritability within the locus (h2 = 0.001, 0.005, or 0.01, shown on the horizontal axis of each panel). Effect size of causal SNPs are randomly assigned by *β*_i_ = N(0,1) and then adjusted based on given heritability. The curves represent Receiver Operating Characteristic (ROC) curve averaged across 50 different simulations with corresponding standard error.

Using posterior causal probabilities of each SNP to be causal (q_i_), we evaluated the power of detecting the actual causal variants and obtained the corresponding Receiver Operating Characteristic (ROC) curve for three methods, and finally calculated area under these curves (AUC) for comparison. The AUC values of these methods are presented in Fig 2A (simulations where *β*_i_ = 1 for causal SNPs) and Fig 2B (simulations where *β*_i_ = N(0,1) for causal SNPs). Note that these values in the figures are the averaged values of 50 different experiments. As can be seen in Fig 2, Finemap-MiXeR either outperforms the other methods in different scenarios especially for lower heritability/higher polygenicity or has similar performance as other methods. The mean of the AUC values of all those experiments in Fig 2A (mean of the 5x3x3 = 45 different configurations presented in Fig 2A) for Finemap-MiXeR, SuSiE RSS and FINEMAP are 0.870, 0.851 and 0.856, respectively. These values for Fig 2B are 0.819, 0.802 and 0.808, respectively. The corresponding Area Under Precision Recall Curves (AUPRC) are also presented in Figs C and D in S1 Text.

The performance of the variation of our method (Finemap-MiXeR-PCA) was plotted in Fig A in S1 Text. As can be observed from this figure, its performance is quite similar to Finemap-MiXeR’s. Furthermore, in this figure, we also compared the performance of our method when the hyperparameters are known and given by the user or when these hyperparameters are optimized using the corresponding derivatives. As given in this figure, the performance of our methods (Finemap-MiXeR and Finemap-MiXeR-PCA) is almost same if the hyperparameters are also optimized within the algorithm.

We also plotted one to one comparison of the posterior causal probabilities obtained from Finemap-MiXeR and SuSiE RSS using scatter plots presented in Fig B in S1 Text. As seen from this figure, SuSiE RSS and Finemap-MiXeR may have different posterior causal probabilities for several SNPs. On the other hand, as can be seen the histogram of causal and non-causal SNPs in Fig B in S1 Text, their distributions are similar. More importantly, Finemap-MiXeR was able to detect some causal SNPs that are not detected by SuSiE RSS (or other methods which provides similar posteriors as SuSiE RSS). Given the fact that Finemap-MiXeR, SuSiE RSS and FINEMAP have similar accuracy in terms of detecting causal SNPs, it is valuable to identify causal SNPs that may not been detected by other methods. In particular, in these experiments, 7.2 percent of causal SNPs is only detected by Finemap-MiXeR (and not by SuSiE RSS) while 4.2 percent is only detected by SuSiE RSS (and not by Finemap-MiXeR). These numbers for SuSiE RSS and FINEMAP are relatively low, 1.2 and 1.1 percent respectively. Therefore, having such diversity in posterior causal probabilities might suggest using Finemap-MiXeR and SuSiE RSS (or other methods) together to detect more possible causal SNPs.

**Fig 3:**
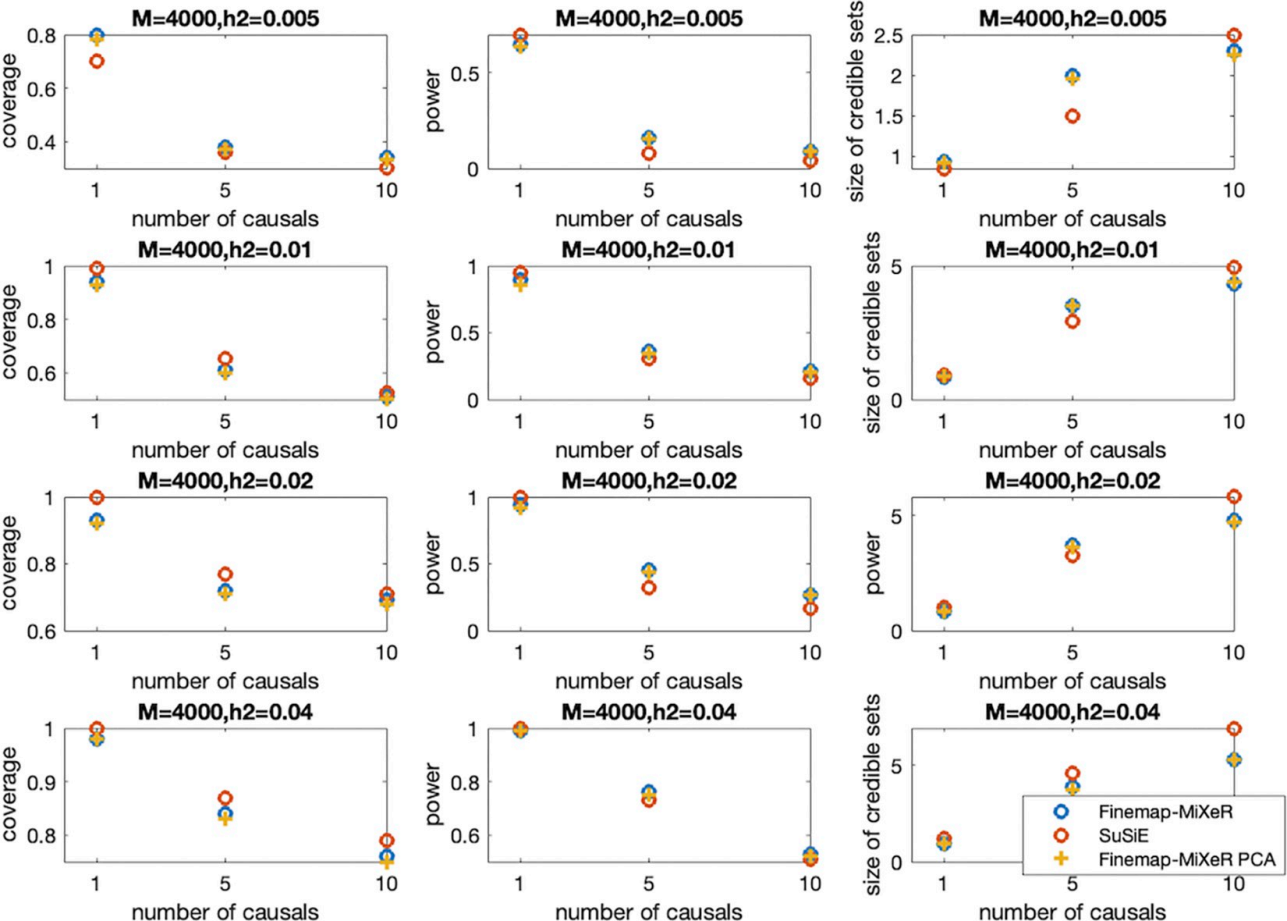
Credible Sets coverage and power comparison of Finamep-MiXeR and SuSiE across different scenarios, varying the true number of causal variants (k = 1, 5, or 10, shown in columns), and the true heritability within the locus (h2 = 0.005, 0.01, 0.02 or 0.04, shown on the horizontal axis of each panel) with M = 4000 SNPs (the size of the locus being analyzed). The effect sizes of causal SNPs are randomly assigned by *β*_i_ = N(0,1) and then adjusted based on the given heritability. The first column corresponds to coverage, which is the probability of a credible set to include at least one causal SNP (and it is equivalent to 1-False Coverage Rate), while the second column gives the corresponding power, i.e. the total proportion of causal SNPs detected by all reported credible sets. The third column gives the average size (number of variants) in credible sets. η is chosen as 0.5 and q_thr_ = 0.95, both as suggested in SuSiE.

We have also examined the performance of credible sets and compared it with SuSiE RSS’s credible sets in different metrics. One metric is coverage which is the probability of a credible set includes at least one causal SNP. Other metric is power which is the total proportion of causal SNPs detected by all reported credible sets. Using the similar simulation procedure described in this section, we have examined these metrics in different regimes that are changing by heritability and polygenicity. As can be seen in Fig 3, SuSiE RSS has slightly better coverage results than Finemap-MiXeR in some scenarios. On the other hand, Finemap-MiXeR mostly detect more causal SNPs and thus have higher power values compared to SuSiE RSS. Furthermore, one can observe from Fig 3 that as the heritability (h^2^) decreases and/or the number of causal (k) increases, the power and the coverage performance of both methods decrease. This is an expected behavior and can also be observed from the AUC performance in Fig 2. When heritability is lower or the number of causals is higher, the signal per causal variant is reduced, and this makes it harder to detect causal variants. We can illustrate this by the scenarios in the second row of Fig 3 (h^2^ = 0.01): When k = 10, the power of the Finemap-MiXeR and SuSiE are 0.21 and 0.17, respectively, and corresponding coverage are 0.50 and 0.51, respectively. This implies that each method was able to detect around 20% of the causal variants in credible sets (which is equivalent to detecting 2 causal variants out of k = 10) with a coverage 0.5. On the other hand, as can be seen in the fourth row of the Fig 3, when the heritability is higher (h^2^ = 0.04), the power for k = 10 increases to 0.53 and 0.51 and coverage increases to 0.76 and 0.79, respectively.

### Application to UKB height data

We used UK Biobank (UKB) genome data (N_total_ = 337 145 after QC) and standing height as phenotype to evaluate the performance of the Finemap-MiXeR method using real data. UK Biobank data was obtained under accession number 27412. Our UKB data included 12,926,669 SNPs and 337,145 subjects, derived from the UKB imputed v3 dataset. During sample QC, we selected unrelated individuals with white British ancestry, removed sex chromosome aneuploidy, and excluded participants who withdrew their consent. SNP-based QC was applied as follows: “plink–maf 0.001–hwe 1e-10–geno 0.1”, in addition to filtering SNPs with imputation INFO score below 0.8 and excluding SNPs with duplicated RS IDs. Since the ground truth causal variants for height are not known, we compared the three methods by predicting height using the SNPs finemapped by each of the algorithms, and then evaluating the correlation between the predicted height and the actual height.

Since the main purpose of finemapping is not phenotype prediction, corresponding prediction performance may not be considered as an ultimate metric to compare the accuracy of finemapping methods. On the other hand, for highly polygenic and heritable phenotypes such as height, ground truth causals may not be well known and thus it can still be interesting and useful to compare methods with respect to the predicted performance of finemapped SNPs.

To achieve this, we split the individual-level UKB data into 80% for training, and 20% for testing. Training set was used to perform finemapping and estimate corresponding weight of finemapped SNP in a linear predictor estimating the height; testing set was used to estimate the height and evaluate the correlation with measured height.

We conducted this procedure for multiple loci associated with height. In particular, we chose the loci that are strongly associated with height based on their corresponding p-value of lead SNPs using recent height GWAS [28]. We examined 31 loci whose lead SNPs’ p-value was lower than 10^−60^ and locus size was lower than 10,000. Note that those loci vary in h2 and M (for details see Table A in S1 Text). In order to get input data for the methods, we applied GWAS to those loci using training set and obtained corresponding z-scores. Then using these z-scores, we ran the 3 algorithms (Finemap-MiXeR, SuSiE RSS and FINEMAP) and obtained the posterior causal probability for each SNP.

Afterwards, for each method, we used the SNP with highest posterior causal probability to estimate height using Multiple Linear Regression (MLR). Basically, we estimated the effect size coefficient of this finemapped SNP, using train data and then applied this coefficient to test data to evaluate the performance.

For applying the procedure defined above to UKB data, firstly we excluded covariates that are effective on height. Once we extracted genotype (G) and phenotype data (y) from UKB, we eliminated the effects of covariates (C) such as age, sex, and first 40 principal components. To achieve this, we first fit C and y using MLR and reduce the effect of C from phenotype as

$$\hat{b}={{(C}^{T}C)}^{-1}C^{T}y\equiv C^{+}y$$

$$y_{res}=y-C\hat{b}$$

where y_res_ corresponds to a Covariates-free residual phenotype vector. In this real analysis, we used this vector and corresponding G matrix and applied GWAS to obtain z-scores to run these three finemap methods.

**Fig 4:**
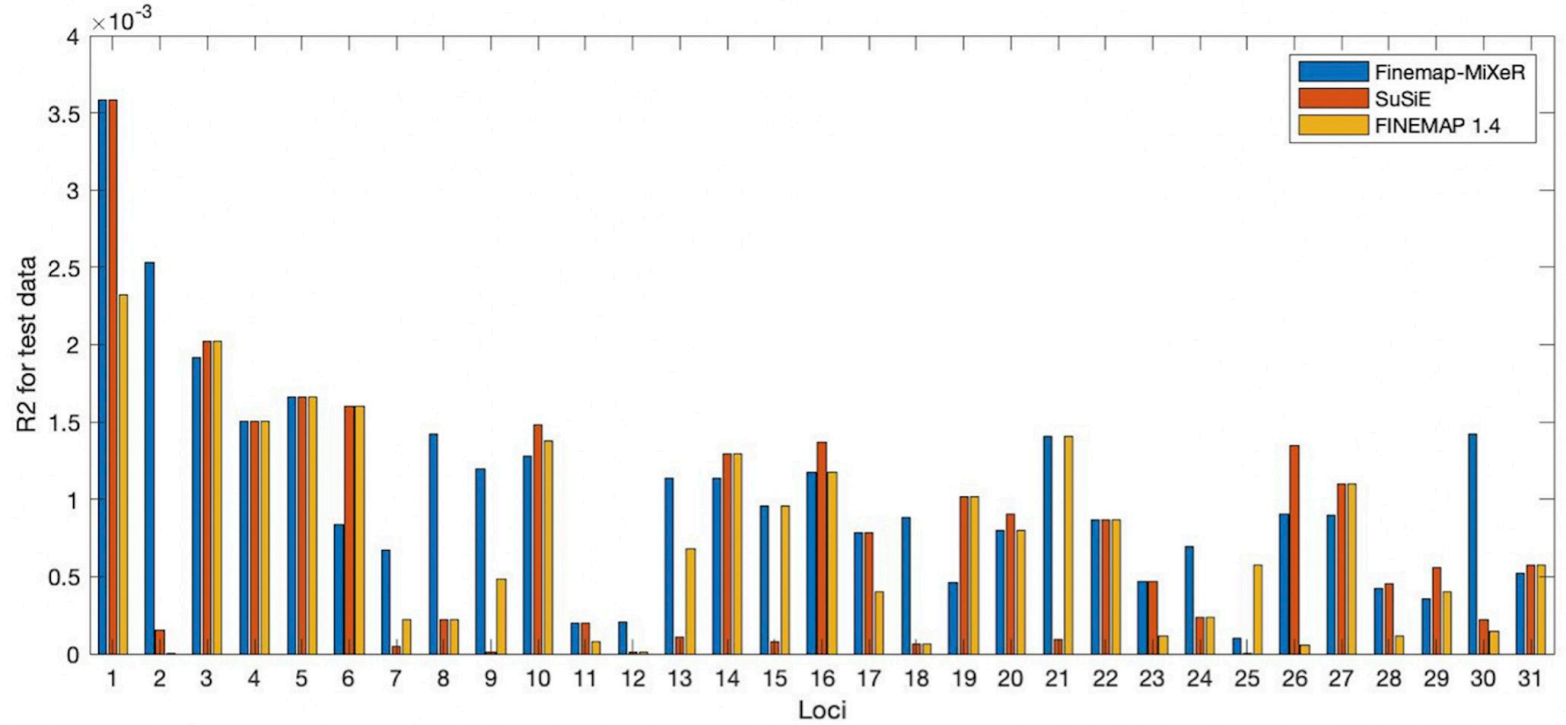
R2 comparison of Finemap-MiXeR with SuSiE and FINEMAP for 31 different loci for UKB height analysis. The details of loci are given in Table A in S1 Text. R2 values corresponds to the correlation between estimated phenotype of test data using the three methods and actual test data. Tools are applied on training data and SNP with highest posterior causal probability were obtained. Then this SNPs is used to estimate test phenotype data. There were 9 loci (29%) [2,7–9,12,13,17,23,29] where Finemap-MiXeR obtained substantially higher R2 than both of the other methods. For 16 loci (51%) [1,3–5,7,10,11,14–16,19–22,27,30], Finemap-MiXeR obtained best (or quite close to the best) R2 results as one or both of the other methods. There were only 6 loci [6,18,24–26,28] where one of the other methods were better than Finemap-MiXeR, with two loci (6%) [25,28] for SuSiE and one locus (3%) [24] for FINEMAP and three locus for both [6,18,26].

As stated above, we examined the loci whose lead SNPs have the lowest p-values. We ran these three algorithms for these loci and get corresponding posterior causal probability (q) for each SNP. Afterwards, we picked the column of G matrix that corresponds to the SNP prioritized by each method, namely G_1_ to calculate the effect size of the finemapped SNPs as

$$w=G_{1}^{+}y_{res}.$$

where $G_{1}^{+}$ is the pseudoinverse of G_1_. We already split the actual data, using N = 0.8 x N_total_ for training, and the rest for testing. In other words, *w* was calculated using training data, and then it was used to estimate the covariate-free test phenotype data, y_test_, by *w* x G_test_, followed by a comparison of the performance on estimation of the phenotypes by 3 methods. We calculated R2 of this estimation, and actual phenotype, as:

$$R2={\left[corr(y_{test},wG_{test})\right]}^{2},$$

and used R2 metric to compare the performance of the methods. The results are presented in Fig 4. As seen from this figure, Finemap-MiXeR was able to detect more predictive SNPs in many loci. In particular, among these 31 loci, there were 9 loci that Finemap-MiXeR obtained substantially higher R2 than both the other methods. For 16 loci, Finemap-MiXeR still provide superior results similar as one or both of the other methods. There were only 6 loci that either of other methods outperformed Finemap-MiXeR significantly, with 1 locus for FINEMAP and 2 loci for SuSiE RSS and 3 loci for both.

### Application to Alzheimer’s disease in 19p13.3/ABCA7

**Fig 5:**
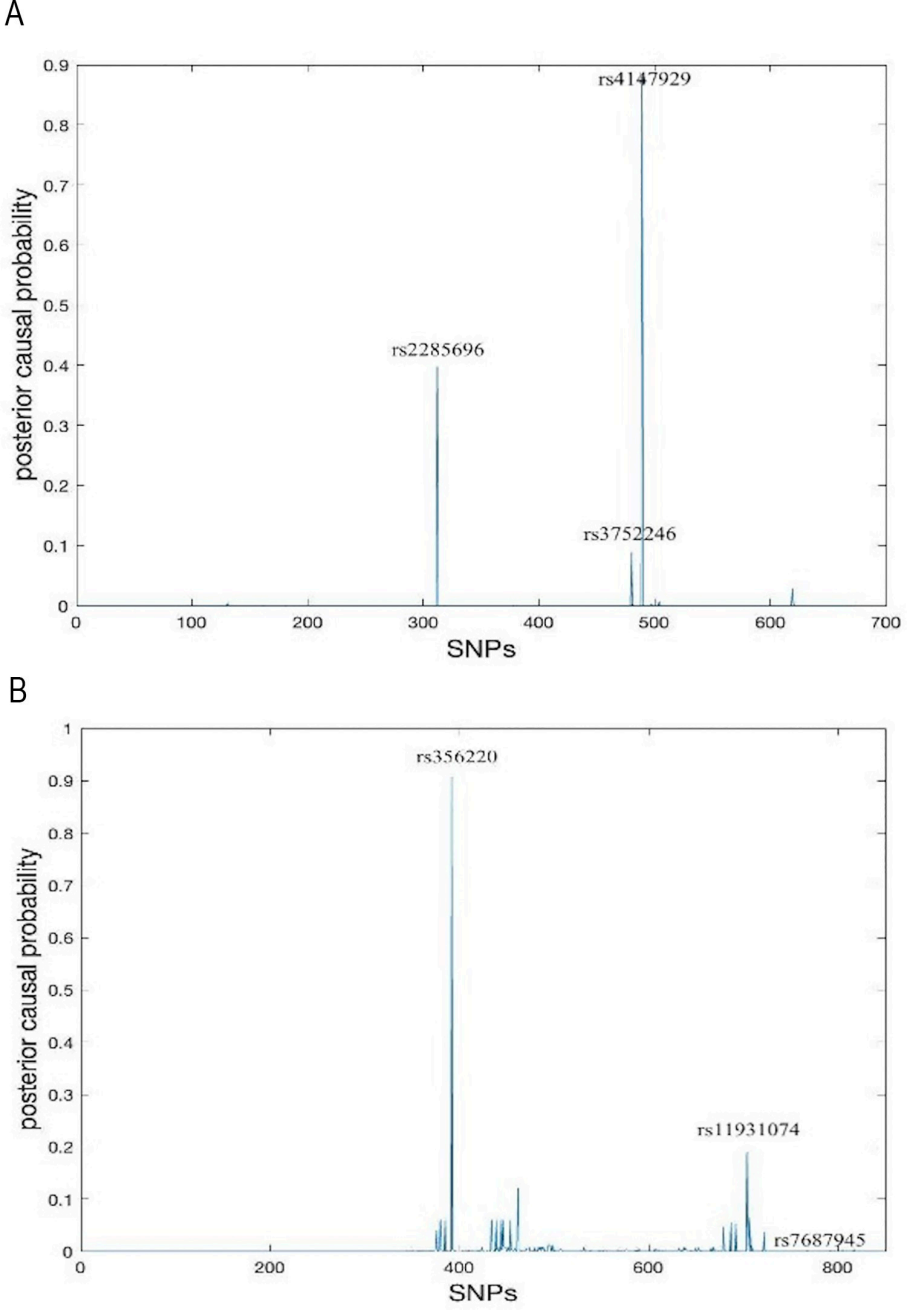
**(A)** Posterior causal probabilities of the variants in 19q13.32 around *rs4147929* variant for ALZ. We used z-scores of this locus in 1 megabase region centered by *rs4147929* variant using summary statistics given in [30]. For A matrix we are using UKB data presented in “Application UKB Data” section. **(B)** Posterior causal probabilities of the variants in 19q13.32 around *rs356220* variant for PD. We used z-scores of this locus in 1 megabase region centered by *rs356220* variant using summary statistics given in [33]. For A matrix we are using UKB data presented in “Application UKB Data” section.

The apolipoprotein E (APOE) gene on chromosome 19q13.32, was the first, and by far the strongest, genetic risk factor for ALZ. Additional signals associated with ALZ have been located in chromosome 19, such as ABCA7 gene in 19p13.3 [29]. Here, we examined this locus to check if our Finemap-MiXeR method is able to detect ALZ associated **rs4147929** variant in this locus. For this aim, we are using summary statistics presented in [30]. We have used the corresponding z-scores in locus 19p13.3. Specifically, we extracted z-scores of this locus in 1 megabase region centered by **rs4147929** variant. We also need to have A matrix (which is the weighted version LD matrix as defined before). For A matrix we are using UKB data presented in “Application UKB Data” section. Using this A matrix and z-scores we have run Finemap-MiXeR and obtained the posterior causal probabilities of the locus as presented in Fig 5A. As shown in this figure, our method was able to detect causal variant **rs4147929** successfully.

### Application to Parkinson’s Disease in 4q22, detection of rs356220 and rs11931074

Previous association studies showed that there is a strong association with Parkinson’s disease (PD) in the 4q22 region [31]. Strongest association in this locus has been detected as **rs356220** in many studies [32]. This locus has also been used as an application in FINEMAP paper and it was aimed to finemap **rs356220** with an additional SNP (rs7687945) that had been detected significant after a conditional analysis done by authors. Here we are aiming to finemap same locus using summary statistics obtained from [33]. We have examined a 1 megabase region centered by **rs356220** and used the same procedure to obtain A matrix.

As can be seen from Fig 5B, our method was able to detect variant **rs356220** as FINEMAP did. On the other hand, our method did not detect rs7687945 as FINEMAP did but detected another variant **rs11931074.** Note that the association of variant **rs11931074** has also been identified recently in some studies [34]. Therefore, our method detects two variants (with highest posterior causal probabilities) that have already been validated in independent studies.

### Runtime performance and computational complexity

We examined the computational complexity of our methods with FINEMAP, SuSiE and SuSiE-RSS using runtime performance of the methods. As presented before, our Finemap-MiXeR method requires O(M^2^) computations per iteration. We also showed that we can reduce the complexity from O(M^2^) to O(p_c_M) by preserving accuracy, where p_c_ << M. In SuSiE, the number of computations per iteration is O(kMN), and in its extension SuSiE-RSS, it is O(kM^2^). In FINEMAP, the worst-case computation required per iteration is O(k^2^M). However, the algorithm was optimized to perform the search only among the SNPs with non-negligible posterior probabilities of being causal, using a hash table in order not to recalculate the same configurations. Thus, the complexity is expected to be reduced when the signal (heritability) is low.

We examined the runtime performance of Finemap-MiXeR, SuSiE and FINEMAP using the same data with different parameters. It is important to note that runtimes may largely differ due to different implementation (FINEMAP 1.4 software used C++ code and is distributed as pre-compiled executable, SuSiE is an R package, Finemap-MiXeR is implemented using MATLAB). On the other hand, we can still compare how the runtime scales with respect to k, M, and h^2^ parameters. It is worth noting that the the computational performance of the methods Finemap-MiXeR, FINEMAP and SuSiE RSS are independent of N, since they use summary statistics, while SuSiE requires individual-level data, hence its computational complexity depends on N. For comparison, previously defined synthetic data created by hapgen2 (N = 10 000) are used. All tools are run in HPC with Intel Xeon CPU E5-2698 v4 @2.20GHZ.

**Fig 6:**
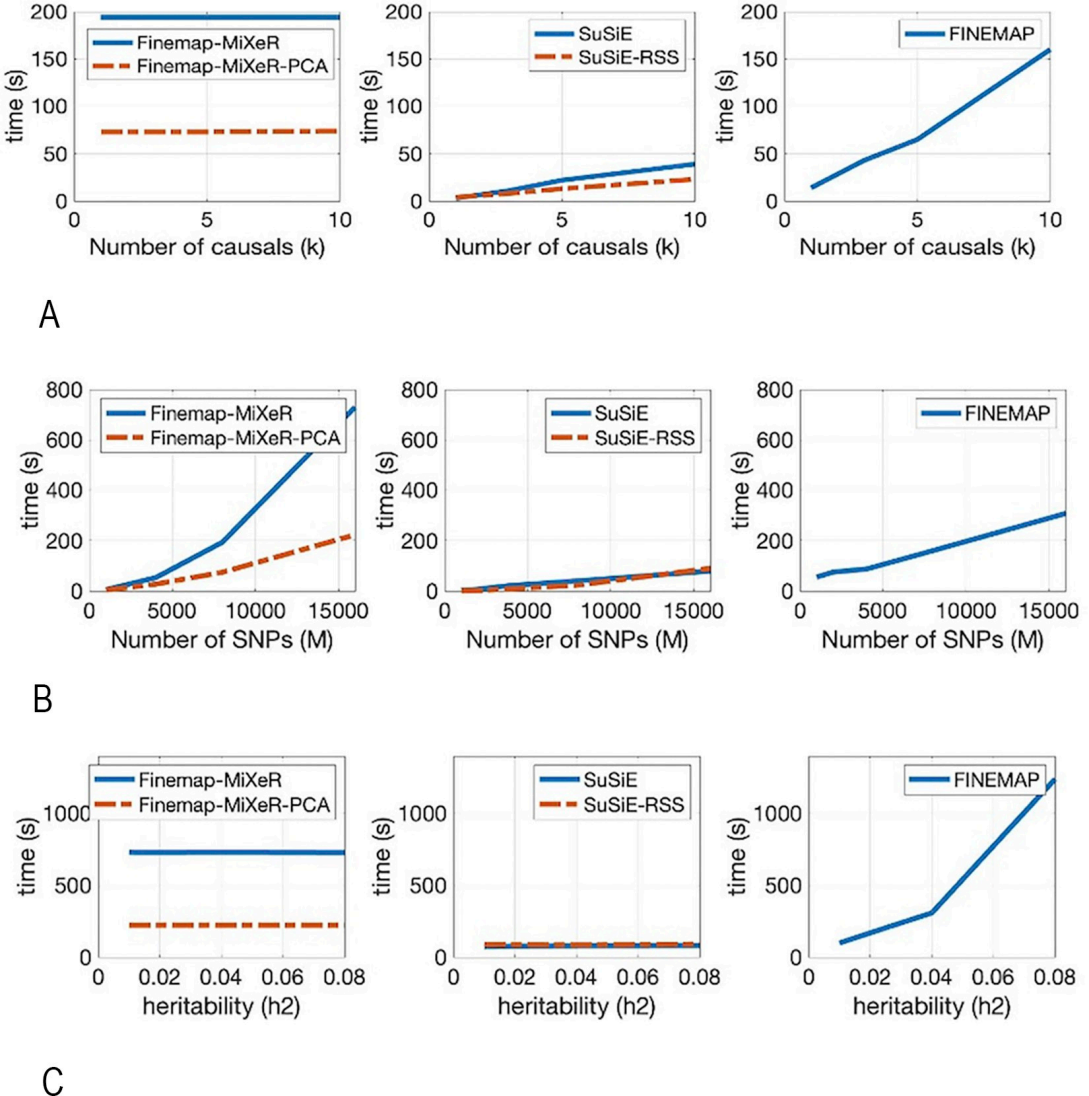
Runtime comparison (in seconds) of the three methods (Finemap-MiXeR, SuSiE, FINEMAP) and two modifications (Finemap-MiXeR-PCA and SuSiE-RSS). Note that these figures are obtained using the synthetic data described in “Simulation with Synthetic data” section. First row of figures: varying size of number of causals from 1 to 10 while keeping constant M and h2. Second row of figures: varying size of the locus M from 1000 to 16000 while keeping constant true heritability (h2 = 0.04) and true number of causals (k = 10). Third row of figures: varying size of the true h2 while keeping M = 16000 and k = 10 as constant. (**A**) Computational performance on varying size of number of causals(k) from 1 to 10 while keeping M and h2 as constant. (**B**) Computational performance on varying size of the locus (M) from 1000 to 16000 while keeping h2 and k as constant. **(C)** Computational performance on varying size of the true h2 from 0.01 to 0.08 while keeping M and k as constant.

As can be seen in Fig 6, for Finemap-MiXeR, the required running time increased as the square of M. Similarly, for SuSiE-RSS, it increased as the square of M, but it also scaled linearly with k. In SuSiE, the runtime was proportional to M and N and higher compared to SuSiE-RSS when N<M, but when M increased, SuSiE was faster than SuSiE-RSS as expected. On the other hand, the FINEMAP runtime increased directly proportional with M, but was more sensitive to the increase in h^2^ (which is an expected behavior as explained above). Furthermore, in SuSiE, SuSiE-RSS and FINEMAP, the runtime increased as the number of causal variants increased, while in Finemap-MiXeR, the number of causal variants did not affect runtime performance. Finally, our extended version of Finemap-MiXeR, Finemap-MiXeR-PCA, reduced the rate of increase of runtime as M increases. This is expected, since computation per iteration is proportional with p_c_M, where p_c_ is typically on the order of 100 and this is generally much lower than the size of a locus, M. Although this method consumes some time to determine eigenvalues and eigenvectors before starting the iteration, it is still much faster than the Finemap-MiXeR and it reduced the rate of increase with M.

## Discussion

Variational Bayesian approach is becoming increasingly popular in statistical genetics due to its flexibility, improved accuracy and computational efficiency compared to other Bayesian methods. In the present study, we used this approach for finemapping, and developed the novel Finemap-MiXeR method.

The Finemap-MiXeR method performs better in terms of accuracy compared to other methods when we conduct comprehensive experiments on synthetic genetic data with different parameters (heritability, number of causal SNPs, loci length). The performance improvements were also observed in applications with real genetic data. To this end, we applied the methods on height, using samples from the UKB. We evaluated multiple loci associated with height, varying in their heritability, loci length and observed that our method outperformed the other methods in most scenarios, yielding better accuracy in predicting the phenotype. Furhermore, we have validated our method on ALZ and PD applications.

One of the main reasons of these improvements in accuracy is MiXeR model’s flexibility in obtaining Bayesian inference for finemapping which leads to more accurate detection of causal variants. While the improvement in terms of accuracy compared to existing methods can be regarded as marginal, we believe that future extensions of the method will yield further improvements in the method’s accuracy. In this paper, we assumed that all SNPs have equal priors and hyperparameters are constant across all SNPs. On the other hand, this assumption would be relaxed, and it is also possible to apply enriched priors to improve method’s accuracy.

Another benefit of Finemap-MiXeR is its computational effectiveness. Thanks to the MiXeR model with our tractable optimization function, our method’s complexity only depends on the size of the locus (M) and does not increase as the number of causals and/or locus’s heritability increase, unlike the other methods do. In particular, although our method’s complexity is increased by O(M^2^) and thus is comparable with SuSiE (O(kMN)) and SuSiE-RSS (O(kM^2^)), our method’s complexity is independent of the number of causals. Furthermore, unlike FINEMAP method, our method´s computational complexity is independent from the heritability. Finally, using Finemap-MiXeR-PCA, it is possible to reduce the complexity of our method to O(p_c_ M) hence to make it linearly scalable with M. Furthermore, unlike many other methods, our method does not require to compute the inverse of the LD matrix, which can be problematic due to dimensionality and rank deficiency.

Variational Bayesian approach has been used to improve the accuracy of the polygenic risk scores (PRS), optimizing Evidence Lower Bound (ELBO) using variational Expected Maximization (EM) algorithm [23]. Here, we optimized ELBO using ADAM algorithm instead of the variational EM [23], leading to better accuracy and better computational complexity compared to the existing finemapping methods. Applying variational Bayesian inference in the context of the MiXeR model to estimate posterior effect size distribution of individual SNPs provides broad opportunities for novel applications of this model in statistical genetics. Beyond finemapping, it can be used together with gene set enrichment analysis thus improving functional interpretation of the GWAS findings. Furthermore, our model can be also extended to cross-ancestry and cross-trait finemapping. Particularly, thanks to the flexibility of our optimization procedure, we can use the same framework for further improvements in Finemap-MiXeR tool, increasing its accuracy by leveraging differential enrichment in functional annotations [35], and extending it to other applications, e.g. finemapping causal variants underlying multiple traits [36], or performing cross-ancestry analysis for a single trait. We may utilize our mathematical framework with the existing bivariate-MiXeR model to optimize the corresponding ELBO and to perform finemapping in cross-traits [18], or we may incorporate enriched priors by combining our method with another extension of MiXeR model for the gene-set enrichment called GSA-MiXeR [37]. Furthermore, trying different parametric families for derivation of ELBO might potentially improve the performance further.

Despite of these advantages and promising results, our method has certain limitations. Although our method is computationally efficient and is shown to scale better than other methods with respect to various parameters, the wall runtime is generally slower than SuSiE RSS, due to the difference in implementation and software optimization of the tools. Another point is that our method constructs credible sets after obtaining the posterior causal probabilities. In future studies, we may also use the credible sets concept during the inference such as incorporation of priors with respect to possible credible sets. This, as a future work, would be able to improve the performance and address some existing challenges. For instance, in the current approach, two true causal SNPs may be assigned to the same credible set if they are in high LD. These limitations, however, do not preclude real-world application of our method and its software implementation.

In conclusion, Finemap-MiXeR is a novel and accurate method for finemapping analysis of GWAS data from complex human traits and has strong potential for further extensions.

## Supporting information

S1 Notes Includes all the technical details regarding the derivation of the proposed method. (PDF)

S1 Text Includes more simulation results. (DOCX)
