# Supplementary material for: Finemap-MiXeR: A variational Bayesian approach for genetic finemapping
Source: PLoS Genet. 2024 Aug 15;20(8):e1011372. doi: 10.1371/journal.pgen.1011372 (PMC11349196; doi:10.1371/journal.pgen.1011372)
Supplement: S1 Notes — (PDF) [file pgen.1011372.s001.pdf]

Supplementary Notes

Introduction

Human genetics studies show that the individual genome of a person is linked to his/her observed features, or phenotypes, such as standing height, or the case/control status of heritable disorders such as schizophrenia. In this material, we consider quantitative phenotypes, $y \in \mathbb{R}$, but the results are also valid for binary phenotypes $y \in \{0, 1\}$. The genome of a given person is typically encoded as a vector $\mathbf{g} = (g_1, \ldots, g_M)^T$, formed by M genetic features, typically $g_i \in \{0, 1, 2\}$ indicates the number of reference alleles at a specific genetic locus, such as a single-nucleotide polymorphism (SNP).

Most of the available models assume a linear relationship between the genetic features and the observed phenotype, i.e. assume that there is a vector $\beta = (\beta_1, \ldots, \beta_M)^T$, such that

$$y = \mathbf{g}^T\beta + e = \sum_{i=1}^{M} \beta_i g_i + e,$$

where $\beta_i \in \mathbb{R}$ indicates the effect of i-th genetic variant, and e is the residual contribution of non-genetic (environmental) factors, and non-linear genetic effects. Making inference about β is crucial for our understanding of the genetics of complex human traits, as it may discover novel drug targets and suggest better treatment strategies.

Recent genome-wide association studies (GWAS) collect genotype and phenotype information from large cohorts of individuals. For example, UK Biobank study has released information for $N = 500.000$ individuals, $M = 93.000.000$ genetic variants, and tens of thousands of quantitative and binary phenotypes. To study a phenotype y, e.g. standing height, we have measurements $\mathbf{y} = (y_1, \ldots, y_N)^T$ across all individuals, and a genotype matrix $G = \{g_{ki}\}$, $k = 1, \ldots N$, $i = 1, \ldots M$. Our goal is to solve a system of linear equations $\mathbf{y} = G\beta$, with known $\mathbf{y}$ and G, w.r.t. unknown β.

As both $\mathbf{y}$ and G contain sensitive (individual-level) information, GWAS studies typically keep this information confidential and release the *GWAS summary statistics*. Ignoring all genetic variants except j-th, consider a simple linear regression model $y \sim g_j$. The corresponding regression coefficient $\hat{\beta}_j$ can be estimated as $\hat{\beta}_j = \frac{\mathbf{v_j^T y}}{\mathbf{v_j^T v_j}}$, where $\mathbf{v}_j = (g_{1j}, \ldots, g_{Nj})^T$ is a vector of j-th genetic features across individuals. The standard error of $\hat{\beta}_j$ estimate vary substantially across genetic features, therefore in addition to $\hat{\beta}_j$ GWAS summary statistics provide statistical significance, $z_j = \frac{\hat{\beta}_j}{se(\hat{\beta}_j)}$. It would be more correct to use the notation t_j instead of z_j and call it Wald's t-statistic, but due to large N we usually denote it z_j and refer to it as z-score. Across genetic variants $j = 1, \ldots, M$, we have a vector $\mathbf{z} = (z_1, \ldots, z_M)$ of z-scores, $z_j = \frac{\hat{\beta}_j}{se(\hat{\beta}_j)}$. It is possible to derive that such z-scores are linearly related to β via matrix $A = (a_{ij})$, derived from $G = (g_{ij})$:

$$z_j = \sum_{i=1}^{M} a_{ij}\beta_i + e_j,$$

where $p(e_j) = N(e_j|0, \sigma_0^2)$ and typically $\sigma_0^2 = 1$. The elements of matrix A can be obtained by $a_{ij} = \sum_{i=1}^{M} \sqrt{N\hat{H}_i}\hat{r}_{ji}$ as defined in [1] where $\hat{H}_i$ is the sample heterozy-

gosity of variant i and $\hat{r}_{ji}$ is sample correlation coefficients between variant i and j. The main goal is to solve a system of linear equations $\mathbf{z} = A\beta$, with known $\mathbf{z}$ and A, w.r.t. unknown β, knowing that A is sparse and band matrix.

MiXeR Model

In MiXeR model, we postulate a spike-and-slab prior distribution on β_i:

$$p(\beta_i) = (1 - \pi_1)N(\beta_i|0, 0) + \pi_1 N(\beta_i|0, \sigma_\beta^2),$$

where $\pi_1 \in [0, 1]$ indicates the weight in the mixture, $N(\beta_i|0, \sigma_\beta^2)$ denotes the normal distribution function of β_i with zero mean and σ_β^2 variance (except for a special case $N(\beta_i|0, 0)$ which indicates probability mass at 0), and σ_β^2 corresponds to the variance of non-zero effects and can be obtained from heritability(h^2) as $h^2 = \sigma_\beta^2 \pi_1 \sum_{i=1}^{M} \hat{H}_i$. For now we assume that parameters $\theta = (\pi_1, \sigma_\beta^2, \sigma_0^2)$ are the same across all SNPs, i.e. do not depend on i. It is also possible to do the same analysis with SNP-specific priors but left as a future work.

For finemapping, we introduce latent variables $u_i \in \{0, 1\}$ following Bernoulli distribution, $p(u_i) = Bern(u_i|\pi_1)$ where $u_i = 1$ implies that SNP i is causal and $u_i = 0$ otherwise. Then the full probabilistic model is $p(z, \beta, u|\theta) = p(z|\beta, \theta) \cdot p(\beta|u, \theta) \cdot p(u|\theta)$ and its product components can be written as:

$$\begin{aligned}
&p(z_j|\beta_1, \ldots, \beta_M, \theta) = N\Big(z_j \Big| \sum_{i=1}^{M} a_{ij}\beta_i, \sigma_0^2\Big), \\
&p(\beta_i|u_i = 0, \theta) = N(\beta_i|0, 0), \quad p(\beta_i|u_i = 1, \theta) = N(\beta_i|0, \sigma_\beta^2), \\
&p(u_i|\theta) = Bern(u_i|\pi_1).
\end{aligned}$$

Variational Bayesian inference

A tricky part of the model is that z_j may depend on multiple β_i. After observing $z = (z_1, \ldots, z_M)^T$, we are aiming to do inference on θ by using maximum likelihood:

$$p(z|\theta) = \int_u \int_\beta p(z, \beta, u|\theta) du d\beta \to \max_\theta .$$

Next, for obtaining a tractable optimization function, we use Evidence Lower Bound (ELBO) and, instead of $p(z|\theta) \to \max_\theta$ and optimize its Variational Lower Bound:

$$\begin{aligned}
\log p(z|\theta) = E_{q(\beta,u)}[\log p(z, \beta, u|\theta) - \log q(\beta, u)] + KL(q(\beta, u)||p(\beta, u|z, \theta)) \geq \\
\geq E_{q(\beta,u)}[\log p(z, \beta, u|\theta) - \log q(\beta, u)] = \mathcal{L}(q, \theta) \to \max_{q,\theta},
\end{aligned}$$

where $KL(q(\beta, u)||p(\beta, u|z, \theta))$ is Kullback–Leibler divergence and it is a measure of how distribution $q(\beta, u)$ is different from $p(\beta, u|z, \theta)$. Therefore, choosing $q(\beta, u)$ close to the distribution of $p(\beta, u|z, \theta)$ leads to low values of $KL(q(\beta, u)||p(\beta, u|z, \theta))$ term,

thus making $\mathcal{L}(q,\theta)$ a tight bound of $\log p(z|\theta)$. In this case, the optimization problems $p(z|\theta) \to \max_\theta$ and $\mathcal{L}(q,\theta) \to \max_{q,\theta}$ are almost equivalent (in a sense that any local maximum of the second problem will also yield a local maximum of the original optimization problem). We will search $q(\beta,u)$ from the following a parametric family:

$$q(\beta,u) = \prod_{i=1}^{M} Bern(u_i|q_i)N(\beta_i|\mu_i,\sigma_i^2).$$

Using this model and parametric family, we can optimize $\mathcal{L}(q,\theta)$ and obtain the parameters of the $q(\beta,u)$ which corresponds to the posterior causal probability of each SNP (q_i), and parameter (μ_i) indicating corresponding effect size and its variance (σ_i^2).

To optimize $\mathcal{L}(q,\theta)$, we use ADAM algorithm [2] to explicitly optimize q_i, μ_i and σ_i^2 parameters as well as θ. As you will observe in the next chapters, in order to apply ADAM algorithm, the first derivatives of the objective function are required. To do so, we need to obtain a tractable formula for $\mathcal{L}(q,\theta)$ which can be written initially as;

$$\begin{aligned}
\mathcal{L}(q,\theta) &= E_{q(\beta,u)} \log p(z|\beta,\theta) + E_{q(\beta,u)} \log \frac{p(\beta|u,\theta)p(u|\theta)}{q(\beta,u)} = \\
&= E_{q(\beta)} \log p(z|\beta,\theta) + E_{q(\beta)q(u)} \log \frac{p(\beta|u,\theta)p(u|\theta)}{q(\beta)q(u)} = \\
&= E_{q(\beta)} \log p(z|\beta,\theta) + E_{q(\beta)q(u)} \log \frac{p(\beta|u,\theta)}{q(\beta)} + E_{q(\beta)q(u)} \log \frac{p(u|\theta)}{q(u)} = \\
&= E_{q(\beta)} \log p(z|\beta,\theta) + E_{q(u)q(\beta)} \log \frac{p(\beta|u,\theta)}{q(\beta)} + E_{q(u)} \log \frac{p(u|\theta)}{q(u)} = \\
&= E_{q(\beta)} \log p(z|\beta,\theta) - E_{q(u)} KL(q(\beta)||p(\beta|u,\theta)) - KL(q(u)||p(u|\theta)) = \\
&= E_{q(\beta)} \log p(z|\beta,\theta) - E_{q(u)} \sum_{i=1}^{M} KL(q(\beta_i)||p(\beta_i|u_i,\theta)) - \sum_{i=1}^{M} KL(q(u_i)||p(u_i|\theta)).
\end{aligned}$$

Calculating the derivatives of Variational Lower Bound

In this section, we will obtain the first derivatives with respect to decision variables which are μ_i, σ_i^2, and q_i to optimize $\mathcal{L}(q,\theta)$. Firstly, we need to expand the terms of $\mathcal{L}(q,\theta)$ in order to ease derivative calculation. Assuming z_j's are independent, $E_{q(\beta)} \log p(z|\beta,\theta)$ can be rewritten as $E_{q(\beta)} \log p(z|\beta,\theta) = E_{q(\beta)} \sum_{j=1}^{M} \log p(z_j|\beta,\theta)$ hence $\mathcal{L}(q,\theta)$ can also be represented as

$$\mathcal{L}(q,\theta) = \underbrace{E_{q(\beta)} \sum_{j=1}^{M} \log p(z_j|\beta,\theta)}_{T_1} - \underbrace{E_{q(u)} \sum_{i=1}^{M} KL(q(\beta_i)||p(\beta_i|u_i,\theta))}_{T_2} - \underbrace{\sum_{i=1}^{M} KL(q(u_i)||p(u_i|\theta))}_{T_3} = \tag{1}$$

$$T_1 - T_2 - T_3.$$

Hence we divide $\mathcal{L}(q, \theta)$ into three terms in order to ease the workload for derivative calculation.

To deal with the derivatives of $E_{q(\beta)} \log p(z|\beta, \theta)$ (hence T_1) we need to employ reparametrization trick [3]. In particular, it is easy to compute $\frac{\partial E_{q(a)} f(b)}{\partial b}$, but it's unclear how to compute $\frac{\partial E_{q(a)} f(b)}{\partial a}$ and we have such cases in T_1. The Reparametrization trick allows us to circumvent this issue by using a parametric standard distribution ϵ (in our case, the standard normal distribution can be used for this purpose), and reformulating the function with this parametric function as

$$E_{q(\beta_i|\mu_i,\sigma_i^2)} \log p(z|\beta_i, \theta) = E_\epsilon \log p(z|\beta_i(\epsilon, \mu_i, \sigma_i^2), \theta).$$

Let $\boldsymbol{\epsilon} \in [\epsilon_1 \epsilon_2 \ldots \epsilon_M] \sim \boldsymbol{N(0, I)}$, then reparametrization trick can be applied as

$$E_{q(\beta)} \sum_{j=1}^{M} \log p(z_j|\beta, \theta) =$$

$$E_{\boldsymbol{\epsilon}} \sum_{j=1}^{M} \log p(z_j|\beta_1 = \mu_1 + \sigma_1\epsilon_1, \beta_2, \ldots \beta_i = \mu_i + \sigma_i\epsilon_i \ldots \beta_M, \theta) \equiv E_{\boldsymbol{\epsilon}} \sum_{j=1}^{M} \log p(z_j|\beta = \mu + \boldsymbol{\sigma\epsilon}, \theta). \tag{2}$$

Since $z_j = \sum_{i=1}^{M} a_{ij}\beta_i + e_j$, then we may write its distribution as

$$z_{j|\beta=\mu+\boldsymbol{\sigma\epsilon},\theta} = \sum_{i=1}^{M} a_{ij}(\mu_i + \sigma_i\epsilon_i) + e_j \sim N(z_j|\sum_{i=1}^{M} a_{ij}(\mu_i + \sigma_i\epsilon_i), \sigma_0^2). \tag{3}$$

In order to calculate the gradients of T_1 (hence $E_{\boldsymbol{\epsilon}} \sum_{j=1}^{M} \log p(z_j|\beta = \mu + \boldsymbol{\sigma\epsilon}, \theta)$), it would be beneficial to write down $\log p(z_j|\beta = \mu + \boldsymbol{\sigma\epsilon}, \theta)$ explicitly. As Eq. 3 implies, it can be written as

$$\log p(z_j|\beta = \mu + \boldsymbol{\sigma\epsilon}, \theta) = -\log\left[\sqrt{2\pi\sigma_0^2}\right] - \frac{\left(z_j - \sum_{i=1}^{M} a_{ij}(\mu_i + \sigma_i\epsilon_i)\right)^2}{2\sigma_0^2}. \tag{4}$$

If we plug this expression into T_1, then it is possible to get the following expression:

$$\mathrm{T}_1 = E_{\boldsymbol{\epsilon}} \sum_{j=1}^{M} \log p(z_j|\beta = \mu + \boldsymbol{\sigma\epsilon}, \theta) = E_{\boldsymbol{\epsilon}} \sum_{j=1}^{M} -\log\left[\sqrt{2\pi\sigma_0^2}\right] - \frac{\left(z_j - \sum_{i=1}^{M} a_{ij}(\mu_i + \sigma_i\epsilon_i)\right)^2}{2\sigma_0^2}$$

$= - \sum_{j=1}^{M} E_{\boldsymbol{\epsilon}} \log \left[\sqrt{2\pi\sigma_0^2}\right] - \sum_{j=1}^{M} E_{\boldsymbol{\epsilon}} \frac{\left(z_j - \sum_{i=1}^{M} a_{ij}(\mu_i + \sigma_i\epsilon_i)\right)^2}{2\sigma_0^2}$
$= -M \log \left[\sqrt{2\pi\sigma_0^2}\right] - \sum_{j=1}^{M} E_{\boldsymbol{\epsilon}} \frac{\left(z_j - \sum_{i=1}^{M} a_{ij}\mu_i - \sum_{i=1}^{M} a_{ij}\sigma_i\epsilon_i\right)^2}{2\sigma_0^2}$
$= -M \log \left[\sqrt{2\pi\sigma_0^2}\right] - \sum_{j=1}^{M} E_{\boldsymbol{\epsilon}} \frac{(z_j - \sum_{i=1}^{M} a_{ij}\mu_i)^2 - 2(z_j - \sum_{i=1}^{M} a_{ij}\mu_i)(\sum_{i=1}^{M} a_{ij}\sigma_i\epsilon_i) + (\sum_{i=1}^{M} a_{ij}\sigma_i\epsilon_i)^2}{2\sigma_0^2}.$

If we expand the $E_{\boldsymbol{\epsilon}}$ operator in T_1, we can deduce that the middle term vanishes since it has $E_{\boldsymbol{\epsilon}}[\epsilon_i]$ as a product term;

$$E_{\boldsymbol{\epsilon}}[-2(z_j - \sum_{i=1}^{M} a_{ij}\mu_i)(\sum_{i=1}^{M} a_{ij}\sigma_i\epsilon_i)] = -2(z_j - \sum_{i=1}^{M} a_{ij}\mu_i)(\sum_{i=1}^{M} a_{ij}\sigma_i E_{\boldsymbol{\epsilon}}[\epsilon_i]) = 0, \tag{5}$$

and similarly, we can calculate the first term of T_1 as

$$E_{\boldsymbol{\epsilon}}[(z_j - \sum_{i=1}^{M} a_{ij}\mu_i)^2] = (z_j - \sum_{i=1}^{M} a_{ij}\mu_i)^2. \tag{6}$$

The third term of T_1 is the most challenging part. Firstly we need to rewrite this term as the square of the summation

$$\left(\sum_{i=1}^{M} a_{ij}\sigma_i\epsilon_i\right)^2 = \sum_{i=1}^{M} \left((a_{ij}\sigma_i\epsilon_i)^2 + 2\sum_{k=i+1}^{M} (a_{kj}\sigma_k\epsilon_k a_{ij}\sigma_i\epsilon_i)\right) \tag{7}$$

and then if we employ $E_{\boldsymbol{\epsilon}}$ operator we may get;

$$E_{\boldsymbol{\epsilon}} \left(\sum_{i=1}^{M} a_{ij}\sigma_i\epsilon_i\right)^2 = \sum_{i=1}^{M} \left(E_{\boldsymbol{\epsilon}}(a_{ij}\sigma_i\epsilon_i)^2 + 2E_{\boldsymbol{\epsilon}}\sum_{k=i+1}^{M} (a_{kj}\sigma_k a_{ij}\sigma_i\epsilon_k\epsilon_i)\right), \tag{8}$$

which implies

$$E_{\boldsymbol{\epsilon}} \left(\sum_{i=1}^{M} a_{ij}\sigma_i\epsilon_i\right)^2 = \sum_{i=1}^{M} \left((a_{ij}^2\sigma_i^2 E_{\boldsymbol{\epsilon}}[\epsilon_i^2]) + 2\sum_{k=i+1}^{M} (a_{kj}\sigma_k a_{ij} E_{\boldsymbol{\epsilon}}[\sigma_i\epsilon_k\epsilon_i])\right). \tag{9}$$

Note that $E_{\boldsymbol{\epsilon}}[\epsilon_i^2] = 1$ and $E_{\boldsymbol{\epsilon}}[\sigma_i\epsilon_k\epsilon_i)] = 0$ since $k > i$. Then it is possible to further simplify it as;

$$E_{\boldsymbol{\epsilon}}\left(\sum_{i=1}^{M} a_{ij}\sigma_i\epsilon_i\right)^2 = \sum_{i=1}^{M}\left(a_{ij}^2\sigma_i^2\right). \tag{10}$$

Then T_1 can be written as;

$$\begin{aligned} T_1 =& - M\log\left[\sqrt{2\pi\sigma_0^2}\right] - \frac{1}{2\sigma_0^2}\sum_{j=1}^{M}\sum_{i=1}^{M}\left(a_{ij}^2\sigma_i^2\right) - \frac{1}{2\sigma_0^2}\sum_{j=1}^{M}(z_j - \sum_{i=1}^{M} a_{ij}\mu_i)^2 \\ =& -M\log\left[\sqrt{2\pi\sigma_0^2}\right] - \frac{T_A}{2\sigma_0^2} \end{aligned} \tag{11}$$

where $T_A = \sum_{j=1}^{M}\sum_{i=1}^{M}\left(a_{ij}^2\sigma_i^2\right) + \sum_{j=1}^{M}(z_j - \sum_{i=1}^{M} a_{ij}\mu_i)^2$.

Note that, since our main motivation is obtaining $\frac{\partial\mathcal{L}_{q,\theta}}{\partial\mu_i}$, $\frac{\partial\mathcal{L}_{q,\theta}}{\partial\sigma_i^2}$, $\frac{\partial\mathcal{L}_{q,\theta}}{\partial q_i}$, $\frac{\partial\mathcal{L}_{q,\theta}}{\partial\theta}$. It is always worthwhile to keep in mind that $E_{\boldsymbol{\epsilon}}[\epsilon_i] = 0$ and $E_{\boldsymbol{\epsilon}}[\epsilon_i^2] = 1$. Then we may start by evaluating $\frac{\partial T_1}{\partial\mu_i}$:

$$\begin{aligned} \frac{\partial T_1}{\partial\mu_i} =& - \frac{\partial}{\partial\mu_i}M\log\left[\sqrt{2\pi\sigma_0^2}\right] \\ & - \frac{\partial}{\partial\mu_i}\sum_{j=1}^{M} E_{\boldsymbol{\epsilon}}\frac{(z_j - \sum_{i=1}^{M} a_{ij}\mu_i)^2 - 2(z_j - \sum_{i=1}^{M} a_{ij}\mu_i)(\sum_{i=1}^{M} a_{ij}\sigma_i\epsilon_i) + (\sum_{i=1}^{M} a_{ij}\sigma_i\epsilon_i)^2}{2\sigma_0^2} \\ =& -\sum_{j=1}^{M} E_{\boldsymbol{\epsilon}}\frac{\partial}{\partial\mu_i}\frac{(z_j - \sum_{i=1}^{M} a_{ij}\mu_i)^2 - 2(z_j - \sum_{i=1}^{M} a_{ij}\mu_i)(\sum_{i=1}^{M} a_{ij}\sigma_i\epsilon_i) + (\sum_{i=1}^{M} a_{ij}\sigma_i\epsilon_i)^2}{2\sigma_0^2}. \end{aligned} \tag{12}$$

In Eq. 12, the rightmost term vanishes since it is independent of μ_i and the middle term vanishes since it includes $E_{\boldsymbol{\epsilon}}[\epsilon_i] = 0$. Therefore, it is possible to simplify the expression as

$$\frac{\partial T_1}{\partial\mu_{i^*}} = -\sum_{j=1}^{M} E_{\boldsymbol{\epsilon}}\frac{\partial}{\partial\mu_i}\frac{(z_j - \sum_{i=1}^{M} a_{ij}\mu_i)^2}{2\sigma_0^2} = -\sum_{j=1}^{M}\frac{\partial}{\partial\mu_i}\frac{(z_j - \sum_{i=1}^{M} a_{ij}\mu_i)^2}{2\sigma_0^2} \tag{13}$$

$$= \frac{1}{\sigma_0^2}\sum_{j=1}^{M} a_{i^*j}(z_j - \sum_{i=1}^{M} a_{ij}\mu_i). \tag{14}$$

In a similar manner, it is possible to calculate $\frac{\partial T_1}{\partial\sigma_i}$:

$$\frac{\partial T_1}{\partial \sigma_i} = -\sum_{j=1}^{M} E_{\boldsymbol{\epsilon}} \frac{\partial}{\partial \sigma_i} \frac{(z_j - \sum_{i=1}^M a_{ij}\mu_i)^2 - 2(z_j - \sum_{i=1}^M a_{ij}\mu_i)(\sum_{i=1}^M a_{ij}\sigma_i\epsilon_i) + (\sum_{i=1}^M a_{ij}\sigma_i\epsilon_i)^2}{2\sigma_0^2}. \tag{15}$$

Similar to the previous derivation, one can observe that the first and the second term are vanished by the derivative and expectation operators respectively. Hence the expression can be further simplified as

$$\frac{\partial T_1}{\partial \sigma_{i^*}} = -\sum_{j=1}^{M} \frac{\partial}{\partial \sigma_i^*} E_{\boldsymbol{\epsilon}} \frac{(\sum_{i=1}^M a_{ij}\sigma_i\epsilon_i)^2}{2\sigma_0^2} = -\sum_{j=1}^{M} E_{\boldsymbol{\epsilon}} \frac{\partial}{\partial \sigma_i^*} \frac{(\sum_{i=1}^M a_{ij}\sigma_i\epsilon_i)^2}{2\sigma_0^2} \tag{16}$$

$$= -\sum_{j=1}^{M} E_{\boldsymbol{\epsilon}} 2a_{i^*j}\epsilon_i^* \frac{(\sum_{i=1}^M a_{ij}\sigma_i\epsilon_i)}{2\sigma_0^2}. \tag{17}$$

Note that the purpose of proposing i^* is to avoid confusion with the summation index in Eq. 16. Once we have eliminated this summation, then we will replace it as i. Since ϵ_is are independent and identically distributed, $E_{\boldsymbol{\epsilon}}[\epsilon_i^*\epsilon_i] = 1$ iff $i = i^*$ and 0 otherwise. Using these, we may obtain the following expression

$$\frac{\partial T_1}{\partial \sigma_{i^*}} = -\frac{1}{2\sigma_0^2} \sum_{j=1}^{M} 2a_{i^*j}^2 \sigma_{i^*}. \tag{18}$$

Then using the chain rule $\frac{\partial T_1}{\partial \sigma_{i^*}} = \frac{\partial T_1}{\partial \sigma_{i^*}^2} \frac{\partial \sigma_{i^*}^2}{\partial \sigma_{i^*}}$, we can easily obtain $\frac{\partial T_1}{\partial \sigma_{i^*}^2}$ as

$$\frac{\partial T_1}{\partial \sigma_{i^*}^2} = \frac{-1}{2\sigma_{i^*}} \frac{1}{2\sigma_0^2} \sum_{j=1}^{M} 2a_{i^*j}^2 \sigma_{i^*} = \frac{-1}{4\sigma_0^2} \sum_{j=1}^{M} 2a_{i^*j}^2. \tag{19}$$

It is also quite straightforward to calculate the gradient of T_1 with respect to θ which is $\nabla_\theta T_1 = \begin{bmatrix} \frac{\partial T_1}{\partial \pi_1} \\ \frac{\partial T_1}{\partial \sigma_\beta^2} \\ \frac{\partial T_1}{\partial \sigma_0^2} \end{bmatrix}$.

Since T_1 only depends on σ_0^2 among variables of θ, we may readily calculate it by utilizing (11) as

$$\frac{\partial T_1}{\partial \sigma_0^2} = \frac{T_A - M\sigma_0^2}{2\sigma_0^4}. \tag{20}$$

Hence,

$$\nabla_\theta T_1 = \begin{bmatrix} 0 \\ 0 \\ \frac{T_A - M\sigma_0^2}{2\sigma_0^4} \end{bmatrix}. \tag{21}$$

For T_2, note that it is possible to rewrite it as

$$E_{q(u)} \sum_{i=1}^{M} KL(q(\beta_i)||p(\beta_i|u_i, \theta)) = \sum_{i=1}^{M} E_{q(u)} KL(q(\beta_i)||p(\beta_i|u_i, \theta)). \tag{22}$$

Then, for $u_i = 0$ and $u_i = 1$ we write the expectation explicitly as:

$$\begin{aligned} E_{q(u)} KL(q(\beta_i)||p(\beta_i|u_i, \theta)) &= q(u_i = 0) KL(q(\beta_i)||N(0, \delta^2)) \\ &+ q(u_i = 1) KL(q(\beta_i)||N(0, \sigma_\beta^2)) \\ &= (1 - q_i) KL(q(\beta_i)||N(0, \delta^2)) + q_i KL(q(\beta_i)||N(0, \sigma_\beta^2)) \end{aligned}$$

where δ^2 is a sufficiently small and adjustable parameter to approximate $N(0, \delta^2)$ as Dirac delta function.

In order to obtain the gradients of T_2, the first step would be representing $q(\beta_i)$ and $q(u_i)$ explicitly. Using $q(\beta, u)$, it is possible to write them as

$$\begin{aligned} q(\beta_i) &= N(\beta_i|\mu_i, \sigma_i^2) \\ q(u_i) &= Bern(u_i|q_i). \end{aligned}$$

Then it is possible to evaluate KL divergence in T_2 (and also T_3). In particular, T_2 involves KL divergence of two normal distributions and can be written as [1]

$$\begin{aligned} T_2 = \sum_{i=1}^{M} E_{q(u)} KL(q(\beta_i)||p(\beta_i|u_i, \theta)) = &\sum_{i=1}^{M} (1 - q_i) \left(\log(\frac{\delta}{\sigma_i}) + \frac{\sigma_i^2 + \mu_i^2}{2\delta^2} - \frac{1}{2} \right) + \\ &\sum_{i=1}^{M} q_i \left(\log(\frac{\sigma_\beta}{\sigma_i}) + \frac{\sigma_i^2 + \mu_i^2}{2\sigma_\beta^2} - \frac{1}{2} \right). \end{aligned}$$

[1] Let, x and y be two normal distributions with means μ_1, μ_2 and standard deviations σ_1, σ_2, respectively. Then $KL(x||y) = \left(\log(\frac{\sigma_2}{\sigma_1}) + \frac{\sigma_1^2 + (\mu_1 - \mu_2)^2}{2\sigma_2^2} - \frac{1}{2} \right)$

Then it is straightforward to calculate derivatives as:

$$\frac{\partial T_2}{\partial \mu_i} = \frac{(1-q_i)\mu_i}{\delta^2} + \frac{(q_i)\mu_i}{\sigma_\beta^2}, \tag{23}$$

$$\frac{\partial T_2}{\sigma_i^2} = \frac{1}{2}\left(\frac{(1-q_i)}{\delta^2} + \frac{(q_i)}{\sigma_\beta^2} - \frac{1}{\sigma_i^2}\right), \tag{24}$$

$$\begin{aligned}\frac{\partial T_2}{\partial q_i} &= -\left(\log(\frac{\delta}{\sigma_i}) + \frac{\sigma_i^2+\mu_i^2}{2\delta^2}\right) + \left(\log(\frac{\sigma_\beta}{\sigma_i}) + \frac{\sigma_i^2+\mu_i^2}{2\sigma_\beta^2}\right)\\ &= \log(\frac{\sigma_\beta}{\delta}) - \frac{\sigma_i^2+\mu_i^2}{2\delta^2} + \frac{\sigma_i^2+\mu_i^2}{2\sigma_\beta^2}.\end{aligned} \tag{25}$$

It is clear that there is just one nonzero term in $\nabla_\theta T_2$ and it can be calculated as

$$\frac{\partial T_2}{\partial \sigma_\beta^2} = \sum_{i=1}^{M} \frac{-q_i}{2\sigma_\beta^4}\left(\sigma_i^2 + \mu_i^2 - \sigma_\beta^2\right). \tag{26}$$

Hence

$$\nabla_\theta T_2 = \begin{bmatrix} 0 \\ \sum_{i=1}^{M} \frac{-q_i}{2\sigma_\beta^4}\left(\sigma_i^2+\mu_i^2-\sigma_\beta^2\right) \\ 0 \end{bmatrix}. \tag{27}$$

For the evaluation of $KL(q(u_i)||p(u_i|\theta))$ term in T_3, we need to apply KL divergence of two Bernoulli distributions since $q(u_i) \equiv Bern(u_i|q_i)$ and $p(u_i|\theta) \equiv Bern(\pi_1)$. Then the corresponding KL divergence can be obtained as[2]

$$KL(q(u_i)||p(u_i|\theta)) \equiv KL(Bern(u_i|q_i)||Bern(\pi_1)) = q_i \log\frac{q_i}{\pi_1} + (1-q_i)\log(\frac{1-q_i}{1-\pi_1}).$$

Then,

[2]Let x and y be two Bernoulli distributions with parameters p_x and p_y. Then $KL(x||y) = p_x \log(\frac{p_x}{p_y}) + (1-p_x)\log\frac{1-p_x}{1-p_y}$.

$$T_3 = \sum_{i=1}^{M} q_i \log \frac{q_i}{\pi_1} + (1 - q_i) \log(\frac{1 - q_i}{1 - \pi_1}),$$

and corresponding nonzero derivatives are:

$$\frac{\partial T_3}{\partial q_i} = \log \frac{q_i}{\pi_1} - \log \frac{1 - q_i}{1 - \pi_1}, \tag{28}$$

$$\frac{\partial T_3}{\partial \pi_1} = \sum_{i=1}^{M} \frac{\pi_1 - q_i}{\pi_1 - \pi_1^2}, \tag{29}$$

$$\nabla_\theta T_3 = \begin{bmatrix} \sum_{i=1}^{M} \frac{\pi_1 - q_i}{\pi_1 - \pi_1^2} \\ 0 \\ 0 \end{bmatrix}, \tag{30}$$

hence

$$\nabla_\theta \mathcal{L}_{q,\theta} = \begin{bmatrix} -\sum_{i=1}^{M} -\frac{\pi_1 - q_i}{\pi_1 - \pi_1^2} \\ -\sum_{i=1}^{M} \frac{-q_i}{2\sigma_\beta^4} \left(\sigma_i^2 + \mu_i^2 - \sigma_\beta^2\right) \\ \frac{T_A - M\sigma_0^2}{2\sigma_0^4} \end{bmatrix}. \tag{31}$$

All in all, as summarized in Table 1, we can add up the corresponding derivatives of all terms to get the derivatives of $\mathcal{L}_{q,\theta}$.

	T_1	T_2	T_3	$L_{q,\theta}$
$\partial \mu_i$	$\frac{1}{\sigma_0^2} \sum_{j=1}^{M}$ $a_{ij}(z_j - \sum_{k=1}^{M} a_{kj}\mu_k)$	$\frac{(1-q_i)}{\delta^2} + \frac{(q_i)}{\sigma_\beta^2}$	0	$\frac{1}{\sigma_0^2} \sum_{j=1}^{M} a_{ij}(z_j - \sum_{k=1}^{M} a_{kj}\mu_k)$ - $\frac{(1-q_i)\mu_i}{\delta^2} - \frac{(q_i)\mu_i}{\sigma_\beta^2}$
$\partial \sigma_i^2$	$\frac{-1}{4\sigma_0^2} \sum_{j=1}^{M} 2a_{ij}^2$	$\frac{1}{2}\left(\frac{(1-q_i)}{\delta^2} + \frac{(q_i)}{\sigma_\beta^2} - \frac{1}{\sigma_i^2}\right)$	0	$\frac{-1}{4\sigma_0^2} \sum_{j=1}^{M} 2a_{ij}^2$ - $\frac{1}{2}\left(\frac{(1-q_i)}{\delta^2} + \frac{(q_i)}{\sigma_\beta^2} - \frac{1}{\sigma_i^2}\right)$
∂q_i	0	$\log(\frac{\sigma_\beta}{\delta}) - \frac{\sigma_i^2+\mu_i^2}{2\delta^2} + \frac{\sigma_i^2+\mu_i^2}{2\sigma_\beta^2}$	$\log \frac{q_i}{\pi_1} - \log \frac{1-q_i}{1-\pi_1}$	-$\left(\log(\frac{\sigma_\beta}{\delta}) - \frac{\sigma_i^2+\mu_i^2}{2\delta^2} + \frac{\sigma_i^2+\mu_i^2}{2\sigma_\beta^2} + \log \frac{q_i}{\pi_1} - \log \frac{1-q_i}{1-\pi_1}\right)$
∇_θ	$\begin{bmatrix} 0 \\ 0 \\ \frac{T_A - M\sigma_0^2}{2\sigma_0^4} \end{bmatrix}$	$\begin{bmatrix} 0 \\ \sum_{i=1}^{M} \frac{-q_i}{2\sigma_\beta^4}\left(\sigma_i^2 + \mu_i^2 - \sigma_\beta^2\right) \\ 0 \end{bmatrix}$	$\begin{bmatrix} \sum_{i=1}^{M} \frac{\pi_1 - q_i}{\pi_1 - \pi_1^2} \\ 0 \\ 0 \end{bmatrix}$	$\begin{bmatrix} -\sum_{i=1}^{M} \frac{\pi_1 - q_i}{\pi_1 - \pi_1^2} \\ -\sum_{i=1}^{M} \frac{-q_i}{2\sigma_\beta^4}\left(\sigma_i^2 + \mu_i^2 - \sigma_\beta^2\right) \\ \frac{T_A - M\sigma_0^2}{2\sigma_0^4} \end{bmatrix}$

Table 1: All partial derivatives of $\mathcal{L}_{q,\theta}$

Modifications for Adam Optimization

The direct implementation of ADAM algorithm itself does not take constraints into account. On the other hand our decision variables q_i and σ_i^2, by definition, have to have

Algorithm 1 Modified ADAM algorithm for Finemap-MiXeR

Require: : $\theta = (\pi_1, \sigma_\beta^2, \sigma_0^2)$ (optional) : hyperparameters
Require: : α : Stepsize
Require: : $\beta_1, \beta_2 \in [0, 1)$: Exponential decay rates for the moment estimates, paper parameters are used
Require: : Reparametrize $q_i = \frac{1}{1+e^{-k_f o_i}}$
Require: : $\mathcal{L}(x, \theta)$,: Stochastic objective function with parameters θ where x=[μ σ o]
Require: : $\mathrm{x_0}$: Initial parameter vector $\mathrm{m_0} \leftarrow 0$ (Initialize 1st moment vector)
Require: : $v_0 \leftarrow 0$ (Initialize 2nd moment vector) $t \leftarrow 0$ (Initialize timestep)
  while q_t not converged **do** $t \leftarrow t + 1$
    $g_t \leftarrow \nabla_\theta \mathcal{L}\left(\mathrm{q_{t-1}}, \theta\right)$: (First gradients w.r.t. stochastic objective at timestep t)
    $\mathrm{m_t} \leftarrow \beta_1 \cdot \mathrm{m_t} - 1 + (1 - \beta_1) \cdot \mathrm{g_t}$ (Update biased first moment estimate)
    $\mathrm{V_t} \leftarrow \beta_2 \cdot \mathrm{V_t} - 1 + (1 - \beta_\mathrm{t}) \cdot \mathrm{g_t^2}$ (Update biased second raw moment estimate)
    $\hat{m}_t \leftarrow m_t / \left(1 - \beta_1^2\right)$ (Compute bias-corrected first moment estimate)
    $\hat{v}_\mathrm{t} \leftarrow \mathrm{V_t} / \left(1 - \beta_2^2\right)$ (Compute bias-corrected second raw moment estimate)
    $\mathrm{x_t} \leftarrow \mathrm{x_{t-1}} - \alpha \cdot \widehat{m}_\mathrm{t} / (\mathrm{eps} + \sqrt{\mathrm{vt}}\ )$ (Update parameters)
    if σ_i is smaller than 0, project into (0, ∞)
    Update hyperparameters (θ) in the same manner if it is not given by the user
  end while
    return $\mathrm{x_t}$ (Resulting parameters)

some constraints. In particular, q_i corresponds to the probability of being causal hence it needs to be between 0 and 1. Similarly, since σ_i^2 represents variance, it needs to be non-negative. To satisfy these constraints, we may either employ Reparamtrization (REP) or Projected Gradient (PG) approaches. For optimization of q_i we are using REP by reparametrizing q_i with another variable o_i as

$$q_i = \frac{1}{1 + e^{-k_f o_i}} \tag{32}$$

where k_f is an arbitrarily chosen constant. Therefore, regardless of the optimized value of o_i, q_i is guaranteed to be placed between 0 and 1. Here instead of optimizing with respect to q_i, optimization with respect to o_i is performed by determining the derivative of $\mathcal{L}(q, \theta)$ with respect to o_i and it can be obtain using chain rule;

$$\frac{\partial \mathcal{L}_{q,\theta}}{\partial o_i} = \frac{\partial \mathcal{L}_{q,\theta}}{\partial q_i} \frac{\partial q_i}{\partial o_i}. \tag{33}$$

For σ_i^2, we are using Projected Gradient which is basically projecting the calculated σ_i^2 to the defined space which is (0, ∞) in our case.

For hyperparameters $(\pi_1, \sigma_\beta^2, \sigma_0^2)$, if it is not given by the user, they can also be optimized same manner using the corresponding derivatives. To sum up, we implemented

the ADAM algorithm for finemapping considering all these points as presented in Algorithm 1.

Reducing Computational Complexity with Finemap-MiXeR PCA

As mentioned in the main text, the required computation to calculate derivatives $\frac{\partial \mathcal{L}_{q,\theta}}{\partial \mu_i}$, $\frac{\partial \mathcal{L}_{q,\theta}}{\partial \sigma_i^2}$, $\frac{\partial \mathcal{L}_{q,\theta}}{\partial_i}$ are $O(M^2)$, $O(M)$ and $O(M)$ respectively. Hence, if we can reduce the computation of $\frac{\partial \mathcal{L}_{q,\theta}}{\partial \mu_i}$ somehow, we can also reduce the required computation of the whole algorithm. We present a Principal Component Analysis (PCA) based approach, namely Finemap-MiXeR PCA, to reduce the computational complexity. Firstly let's re-write the exact formula of this derivative in compact form as defined in the main text:

$$\frac{\partial \mathcal{L}_{q,\theta}}{\partial \boldsymbol{\mu}} = \frac{1}{\sigma_0^2}(A_1 + A_2\boldsymbol{\mu})^T - \frac{(1-\boldsymbol{q}) \odot \boldsymbol{\mu}}{\delta^2} - \frac{\boldsymbol{q} \odot \boldsymbol{\mu}}{\sigma_\beta^2}.$$

The problematic part in this expression that requires $O(M^2)$ is $A_2\boldsymbol{\mu}$ which is a MxM matrix and Mx1 vector product where $A_2 = -AA^T$. Let's recall the result of this product as

$$A_p = A_2\boldsymbol{\mu}. \tag{34}$$

Since $A_2 = -AA^T$, the columns of A_2 are highly correlated due to LD structure of matrix A. Therefore, for reducing dimensionality, we may perform Principal Component Analysis (PCA) analysis to A_2 to obtain its eigenvalues and corresponding eigenvectors as

$$cov(A_2) = U\Sigma U^T, \tag{35}$$

where Σ corresponds to diagonal matrix whose diagonal elements are the sorted eigenvalues of cov(A_2) and columns of U is the matrix whose columns are the corresponding eigenvectors. Then we can choose the first p_c eigenvectors of U that covers the pc_{thr}=0.9999 (%99.99) of variation of A_2 as $A_{22} = U^T A_2$ and project A_p into the new dimension as

$$A_{p2} = A_{22}\boldsymbol{\mu}, \tag{36}$$

where A_{p2} is a matrix whose dimensions are p_c x M. If we would like to reconstruct A_p from A_{p2} we can do this by multiplying A_{p2} with U as

$$\hat{A}_p = UA_{p2} = UU^T A_2 \boldsymbol{\mu}. \tag{37}$$

Note that, since U^T and A_2 are fixed, we may precalculate them before the iterations as $B_1 = U^T A_2$ and for each iteration the required calculations are

$$B_2 = B_1 \boldsymbol{\mu}, \tag{38}$$

$$\hat{A}_p = UB_2, \tag{39}$$

where both operations above require O(p_c M) and since p_c is mostly $p_c << M$, the required operations to compute gradients can be reduced importantly by preserving accuracy.

References

[1] A. A. Shadrin, O. Frei, O. B. Smeland, F. Bettella, K. S. O'Connell, O. Gani, S. Bahrami, T. K. Uggen, S. Djurovic, D. Holland *et al.*, "Phenotype-specific differences in polygenicity and effect size distribution across functional annotation categories revealed by ai-mixer," *Bioinformatics*, vol. 36, no. 18, pp. 4749–4756, 2020.

[2] D. P. Kingma and J. Ba, "Adam: A method for stochastic optimization," *arXiv preprint arXiv:1412.6980*, 2014.

[3] M. Titsias and M. Lázaro-Gredilla, "Doubly stochastic variational bayes for non-conjugate inference," in *International conference on machine learning.* PMLR, 2014, pp. 1971–1979.
