# Supplementary material for: Finemap-MiXeR: A variational Bayesian approach for genetic finemapping
Source: PLoS Genet. 2024 Aug 15;20(8):e1011372. doi: 10.1371/journal.pgen.1011372 (PMC11349196; doi:10.1371/journal.pgen.1011372)
Supplement: S1 Text — (DOCX) [file pgen.1011372.s002.docx]

**Fig A.** Area Under the Curve (AUC) comparison for variation of our methods Finemap MiXeR, Finemap-MiXeR-PCA and their extensions with optimizing hyperparameters. Therefore, there are 4 different variations to be presented: Finemap MiXeR, Finemap-MiXeR-PCA, Finemap MiXeR with optimizing hyperparameters, Finemap-MiXeR-PCA with optimizing hyperparameters. Simulations are conducted across different scenarios (as defined in Fig 2a), varying: the size of the locus being analyzed (M=200, 1000, 2000, 4000, or 8000 SNPs per locus, shown in rows); the true number of causal variants (k=1, 5, or 10, shown in columns), and the true heritability within the locus (h2=0.001, 0.005, or 0.01, shown on the horizontal axis of each panel). The curves represent the average AUC values of 50 different simulations with corresponding standard error. The average AUC of those experiments for Finemap MiXeR, Finemap MiXeR with optimizing hyperparameters, Finemap-MiXeR-PCA and Finemap-MiXeR-PCA with optimizing hyperparameters are 0.873, 0.870. 0.868 and 0.865 respectively.


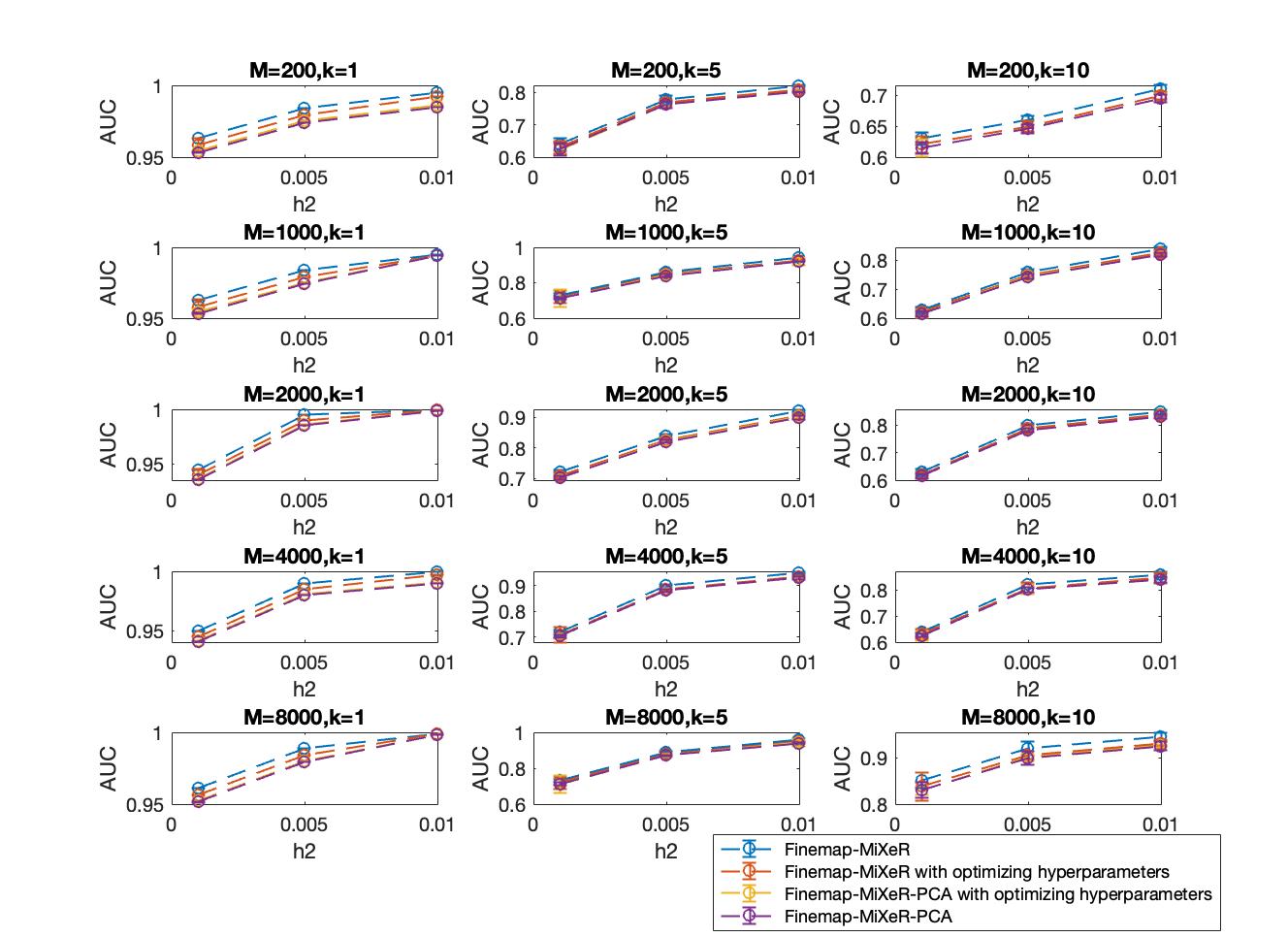
**Fig B.** Comparison of the posterior causal probabilities obtained by Finemap-MiXeR, SuSiE. 1400 different simulations are done for k=1,5 and 10, M=2000 with $h^{2}$= 0,02. Each row presents results with different number of causal, k. Left: The obtained posterior causal probabilities of each SNPs are plotted with respect to other method as scatter plot. The red points correspond to non-causal variants while blue points correspond to causals. Center: Histogram of the posterior causal probabilities of non-causal SNPs. Right: Histogram of the posterior causal probabilities of causal SNPs. Despite Finemap-MiXeR and SuSiE give different posterior causal probabilities for many variants, the distribution of the posterior causal and non-causal SNPs are similar. This also justifies similar accuracy performances.


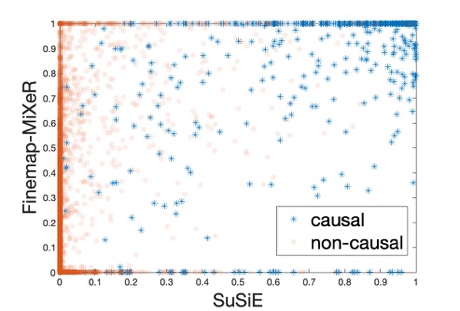

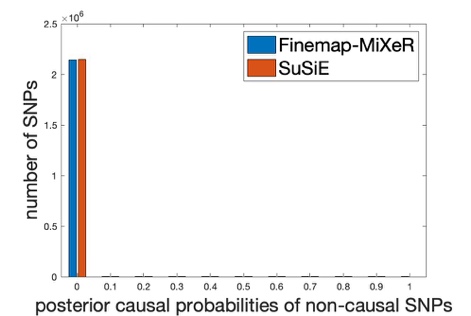

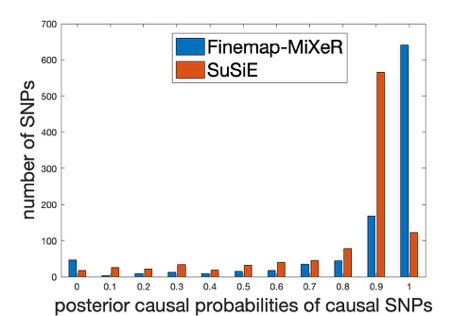


(A) k=1, M=2000 and $h^{2}$=0.02


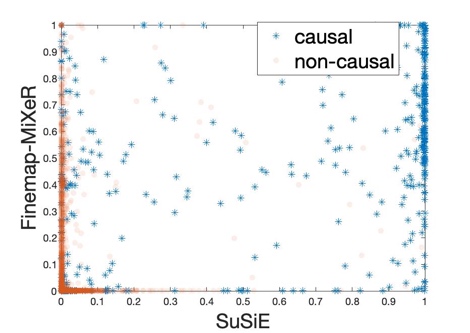

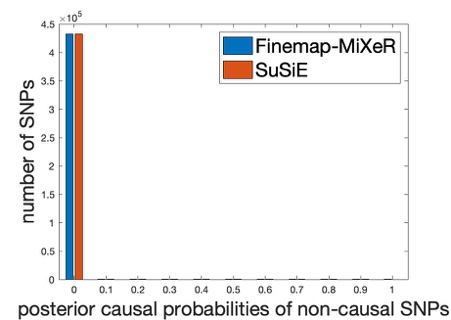

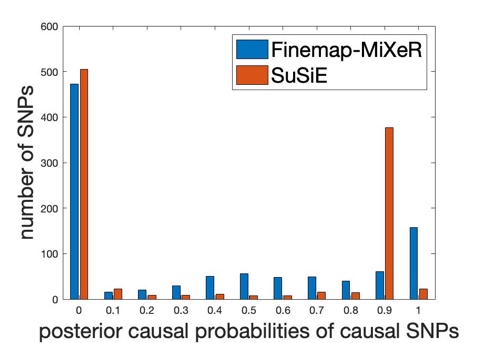


(B) k=5, M=2000 and $h^{2}$=0.02


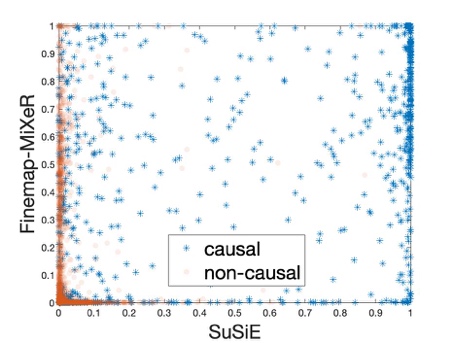

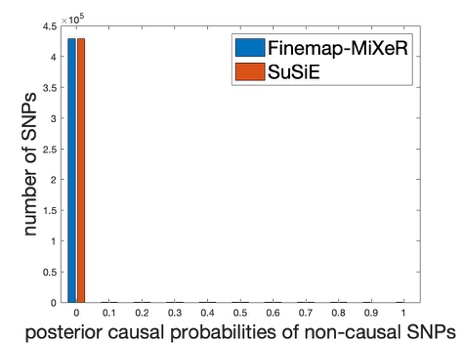

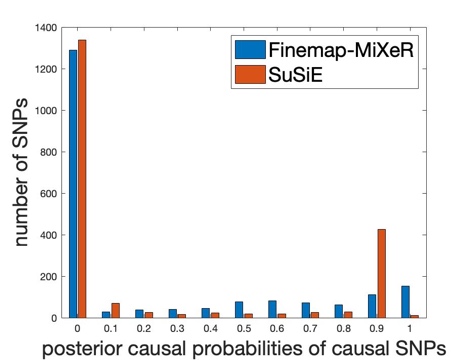


(C) k=10, M=2000 and $h^{2}$=0.02

**Fig C.** Area Under the PR Curve (AUPRC) comparison of Finamep-MiXeR with SuSiE RSS and FINEMAP across different scenarios, varying: the size of the locus being analyzed (M=200, 1000, 2000, 4000, or 8000 SNPs per locus, shown in rows); the true number of causal variants (k=1, 5, or 10, shown in columns), and the true heritability within the locus (h2=0.001, 0.005, or 0.01, shown on the horizontal axis of each panel). Effect size of causal SNPs are randomly assigned as $\beta$_i_=1 and then adjusted based on given heritability. The curves represent Receiver Operating Characteristic (ROC) curve averaged across 50 different simulations with corresponding standard error. The mean values of the AUPRC of Finamep-MiXeR with SuSiE RSS and FINEMAP in these experiments are 0.434, 0.422 and 0.424, respectively.


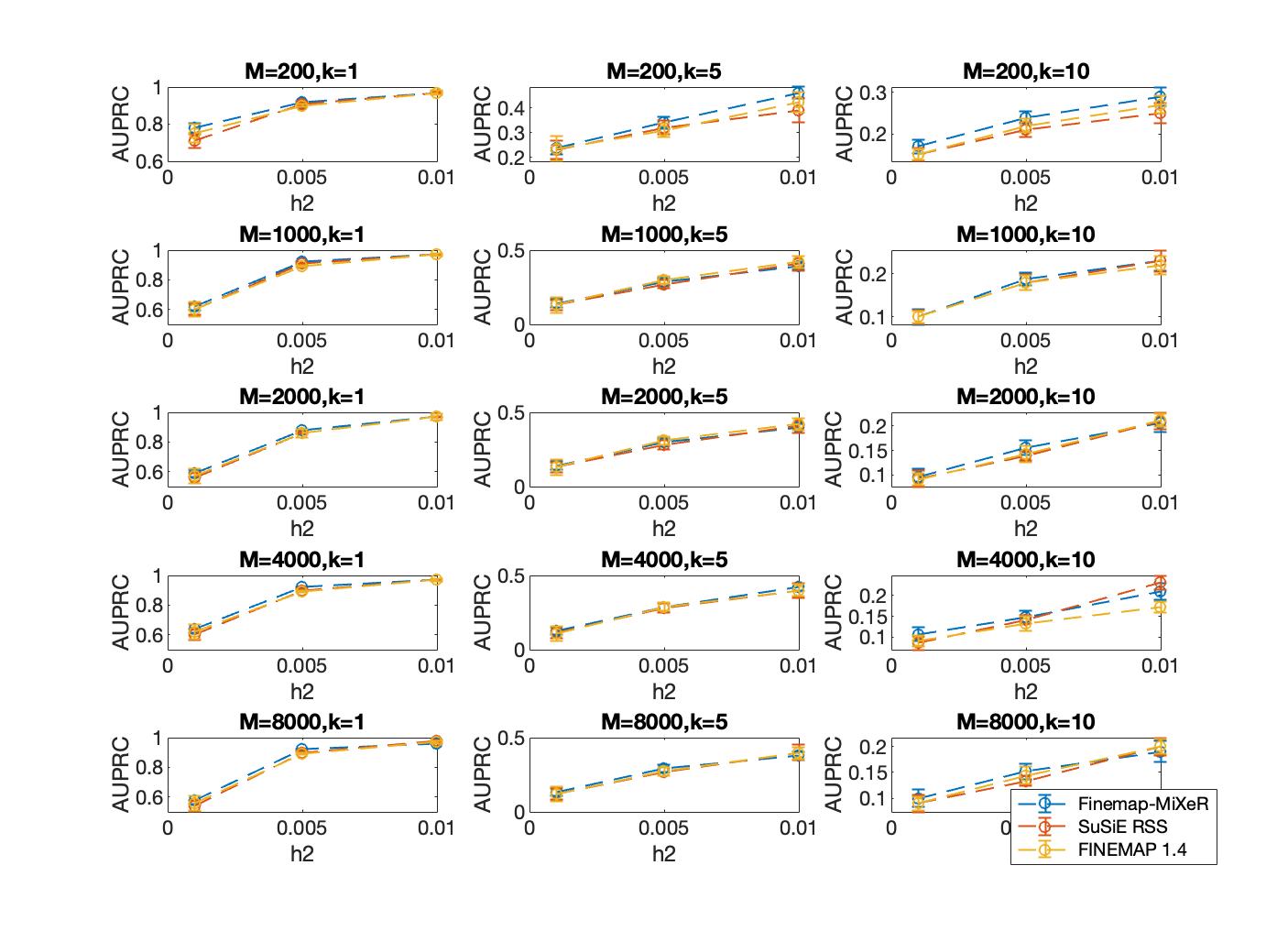


**Fig D.** Area Under the PR Curve (AUPRC) comparison of Finamep-MiXeR with SuSiE RSS and FINEMAP across different scenarios, varying: the size of the locus being analyzed (M=200, 1000, 2000, 4000, or 8000 SNPs per locus, shown in rows); the true number of causal variants (k=1, 5, or 10, shown in columns), and the true heritability within the locus (h2=0.001, 0.005, or 0.01, shown on the horizontal axis of each panel). Effect size of causal SNPs are randomly assigned by $\beta$_i_=N(0,1) and then adjusted based on given heritability. The curves represent Receiver Operating Characteristic (ROC) curve averaged across 50 different simulations with corresponding standard error. The mean values of the AUPRC of Finamep-MiXeR with SuSiE RSS and FINEMAP in these experiments are 0.410, 0.401 and 0.404, respectively.


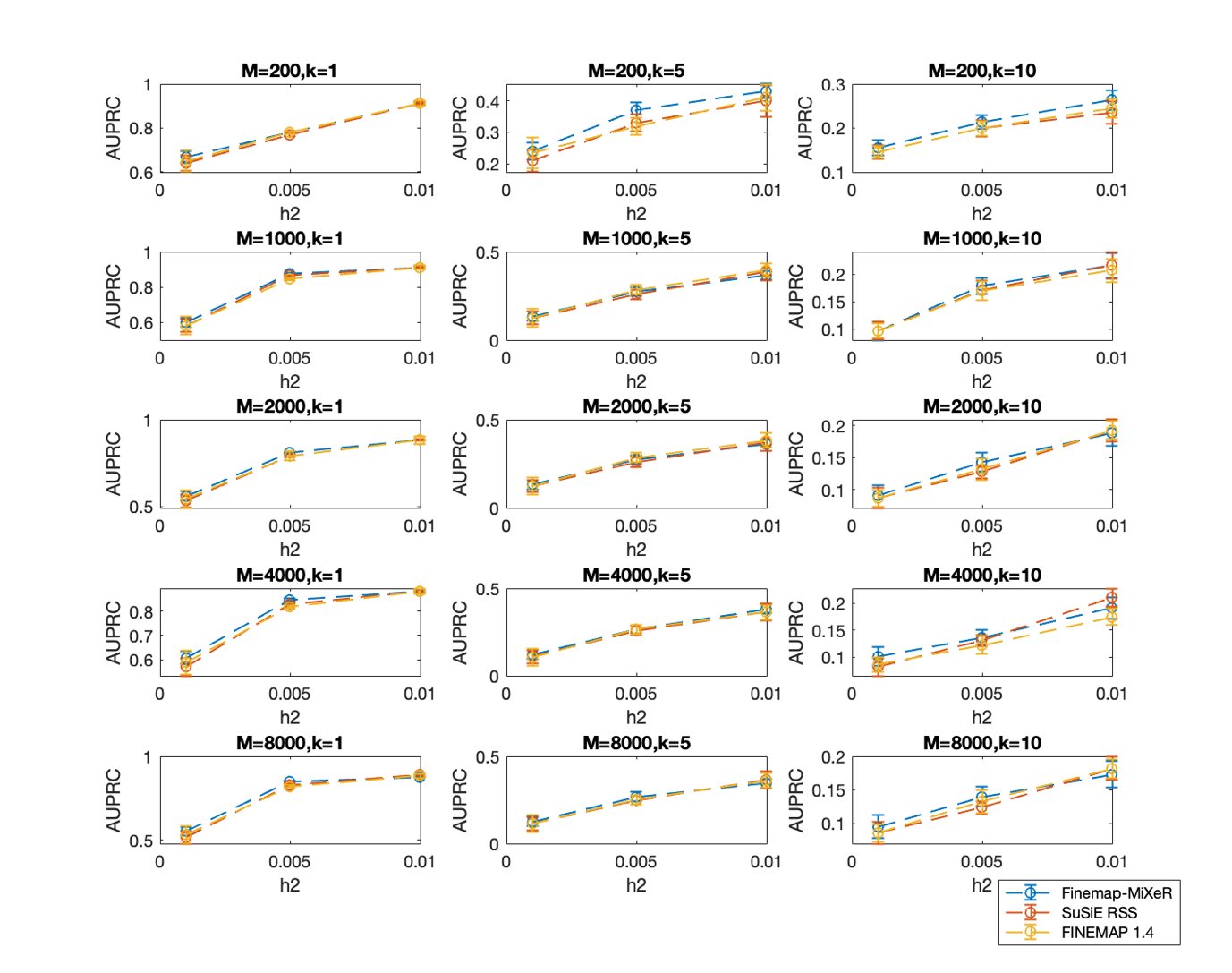


**Fig E.** Sensitivity Analysis of L (the initial number of credible sets) on the power and coverage. The simulations are based on M=4000, h2=0.01 considering 5 true causal variants i.e. k=5 as applied in Figure 3. Our method automatically evaluates L= ⌈ M $\pi_{1}$⌉ and corresponding results are shown as red cross. We also assess the sensitivity of L on coverage and power if L were externally chosen by the user. As seen from these figures, when L is smaller than the true number of causals variants (k=5), power drops as expected since it is possible to miss some true credible sets (L<k). Although there is a slight increase on coverage as L drops this change can be ignored compared to the impact on power. Therefore, starting with a larger L would be preferable to starting with L<k. Nonetheless, our method determines L and hence there is not any need to specify it externally.


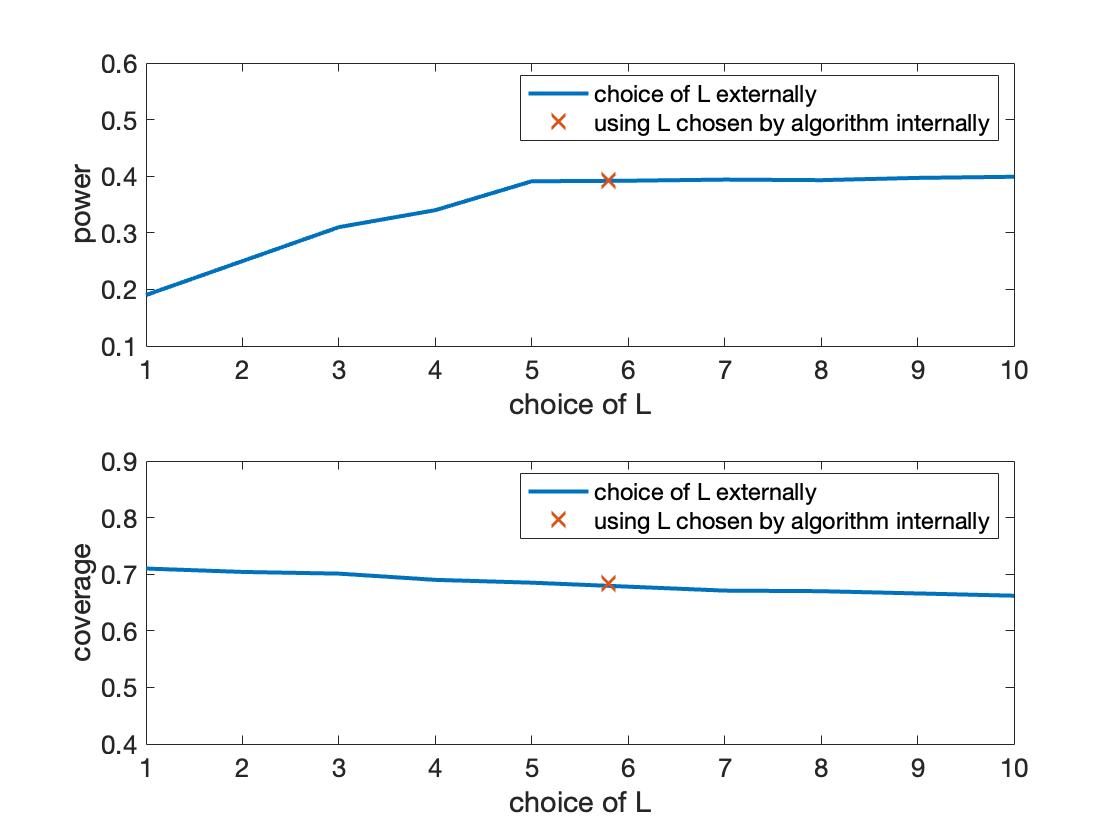


**Table A**. 31 loci associated with height in UKB data are used to estimate R2 values of phenotype (height) estimation. Locus whose lead SNP’s p-value is lower than 10^-60^ and whose size (M) is smaller than 10.000 is selected.

| Locus# | chromosome | Lead SNP | M | Estimated heritability |
| --- | --- | --- | --- | --- |
| 1 | 3 | rs72656010 | 2878 | 0,01530573 |
| 2 | 11 | rs1355603 | 6587 | 0,02923624 |
| 3 | 18 | rs7235010 | 2737 | 0,01254682 |
| 4 | 6 | rs41271299 | 542 | 0,00410876 |
| 5 | 15 | rs72755233 | 3771 | 0,0194017 |
| 6 | 6 | rs9496369 | 2020 | 0,00997987 |
| 7 | 15 | rs28584580 | 2856 | 0,01488572 |
| 8 | 17 | rs368295774 | 3625 | 0,01713786 |
| 9 | 13 | rs3118906 | 2905 | 0,01491732 |
| 10 | 6 | rs1490384 | 1268 | 0,00655242 |
| 11 | 12 | rs76895963 | 3721 | 0,01603232 |
| 12 | 20 | rs1884897 | 1292 | 0,00739424 |
| 13 | 5 | rs244711 | 1864 | 0,00992263 |
| 14 | 6 | rs11243202 | 2307 | 0,01142958 |
| 15 | 6 | rs7740107 | 3229 | 0,01426696 |
| 16 | 8 | rs72656010 | 2462 | 0,01228282 |
| 17 | 15 | rs2871865 | 1072 | 0,00669562 |
| 18 | 3 | rs2194411 | 1339 | 0,00666089 |
| 19 | 1 | rs9435731 | 375 | 0,00228394 |
| 20 | 8 | rs7816131 | 1532 | 0,0071568 |
| 21 | 15 | rs11259936 | 4031 | 0,01875643 |
| 22 | 9 | rs35307904 | 830 | 0,00485567 |
| 23 | 5 | rs11424823 | 1387 | 0,00837447 |
| 24 | 8 | rs4735766 | 1797 | 0,00778689 |
| 25 | 5 | rs7705189 | 2724 | 0,01214858 |
| 31 | 7 | rs798528 | 1224 | 0,00664849 |
| 27 | 3 | rs7652177 | 981 | 0,00522138 |
| 28 | 17 | rs55749333 | 2562 | 0,01203654 |
| 29 | 2 | rs78198962 | 3481 | 0,01666302 |
| 30 | 17 | rs6505216 | 3625 | 0,01713786 |
| 31 | 19 | rs12985850 | 207 | 0,00165972 |
